# Butyrate Ameliorates Insufficient Sleep-Induced Intestinal Mucosal Damage in Humans and Mice

**DOI:** 10.1128/spectrum.02000-22

**Published:** 2022-12-21

**Authors:** Ting Gao, Zixu Wang, Yulan Dong, Jing Cao, Yaoxing Chen

**Affiliations:** a College of Veterinary Medicine, China Agricultural University, Haidian, Beijing, China; b Department of Nutrition and Health, China Agricultural University, Haidian, Beijing, China; University of Nebraska—Lincoln

**Keywords:** sleep deprivation, sleep restriction, gut microbiota, butyrate, college student, small intestines

## Abstract

Insufficient sleep is a key factor in the occurrence of intestinal diseases. This study was performed to clarify how sleep deficiency mediates the intestinal microbiota, metabolite butyrate disturbance induces intestinal mucosal damage, and butyrate ameliorates it. A questionnaire was launched for sleep and intestinal health issues. Twenty-two healthy volunteers were interviewed, and the influence of insufficient sleep on the gut microbiota and metabolite composition was explored. Moreover, a 72-h sleep deprivation (SD) mouse model with or without butyrate supplementation was used to reveal the effect of butyrate on ameliorating small intestines damage caused by SD. The questionnaire survey of 534 college students showed that among 85.39% of the students who slept less than 7 h, 41.76% were suffering from various bowel disorders. High-throughput 16S rRNA pyrosequencing demonstrated that SD and sleep restriction (SR) resulted in downregulation of *Faecalibacterium* and butyrate abundance in the feces of college students. Furthermore, we observed that butyrate supplementation markedly reversed sleep-deprivation-induced small intestinal mucosal injury in mice. Meanwhile, butyrate supplementation inverted the SD-caused inflammation response and oxidative stress and the decline of phospho-glycogen synthase kinase 3β (p-GSK-3β), β-catenin, Nrf2, and cyclin D1 and the increase in histone deacetylase 3 (HDAC3) and phospho-P65 (p-P65) proteins in the small intestines. Furthermore, *in vitro*, the ameliorative effects of butyrate were blocked by treatment with the HDAC3 agonist ITSA-1 and the Nrf2 antagonist ML385 and mimicked by treatment with the HDAC3 antagonist RGFP966 and p-P65 antagonist PDTC. Our study revealed that SD and SR downregulated butyrate production, further causing intestinal homeostasis dysfunction via the HDAC3–p-GSK-3β–β-catenin–Nrf2–NF-κB pathway.

**IMPORTANCE** Radical inflammatory bowel disease (IBD) induced by sleep deficiency is a serious global public health threat. Butyrate, a member of the short-chain fatty acids, exerts multiple effects on it. However, existing research focuses on injury to the colon caused by insufficient sleep, while the changes in the small intestines are often overlooked. This study focused on revealing the influence of insufficient sleep on the intestinal microbiota and its metabolites and further revealed the ameliorative effect of butyrate on sleep deprivation (SD)-induced small intestinal mucosal damage in human and mice. Our studies suggest that butyrate can be used as a probiotic to restore SD-induced IBD and contribute to a better understanding of the mechanisms that govern the beneficial effects of butyrate.

## INTRODUCTION

Long working and commuting times, higher levels of psychological stress, and social and family demands have become in common modern lifestyles, which can lead to severe reductions in sleep duration (1). There are two common types of sleep disorders: sleep deprivation (SD) and sleep restriction (SR). Short-term SD refers to interruptions or reductions in sleep that are shorter than habitual durations, which are commonly seen in shift workers, urban dwellers, surgical patients, etc. (2). In contrast to acute SD, chronic SR refers to sleeping for less than 7 h a day. SR is most common among employees and medical staff, who face high pressure at work, resulting in frequent late working hours. Importantly, sleep deficiency can be a threat factor for ongoing inflammatory bowel disease (IBD) (3–6). Studies have also indicated that genetic defects associated with IBD can lead to accumulation and infiltration of pathogens into intestinal tissues, further promoting dysbiosis and inflammation (7). However, although the rodent model provides insights into the relationship between insufficient sleep and health status, it does not mimic typical real-life human sleep deficiency. Therefore, studying the effects of SD and SR on human intestinal microbiota is necessary to determine their relationship with host intestinal health.

There are a large number of species-rich gut microbiota and their metabolites in the gut, which have been shown to play an important role in regulating gut function and maintaining gut homeostasis (8). Short-chain fatty acids (SCFAs) serve as the primary source of energy for colonocytes and exert major effects on various cell types, including regulation of epithelial gene expression involved in energy metabolism, as well as advance development of mouse intestinal organoids (9, 10). Butyrate is a member of the SCFAs, mainly produced by *Faecalibacterium*, and plays an important role in cell cycle inhibition, immune regulation, and induction of programmed cell differentiation and cell death (11–13). Importantly, Szentirmai et al. indicated butyrate as a vital metabolite of gut bacteria could enhance sleep (14). These phenomena demonstrated the core position of commensal microbiota and their metabolites, including butyrate, in preserving intestinal homeostasis. However, the mechanisms by which SD and SR affect human intestinal microbiota and its metabolites remain unclear. Here, we aimed to determine which intestinal microbiota-derived metabolites participate in regulating intestinal homeostasis in SD and SR. However, most of the current studies pay close attention to the injury of the large intestines, such as colon, from sleep insufficiency, but slight the study of small intestines balance, which has profound influences on various aspects of host physiology, such as metabolic, immune, and endocrine functions (15). The small intestines are the most important part of digestion, and absorption of nutrients and the small intestinal mucosa constitute a vital barrier in the gut against enteric pathogens. Intestinal villi are the main components of the small intestines. Changes in the shape of the villi, such as shortening and ablation, can directly affect the surface area of the villi, which in turn affects the host's ability to absorb nutrients (16), while its strong swing helps to colonize the harmful microbiota. Therefore, exploring the influences of sleep on small intestines health is significant and necessary.

Therefore, in this study, we conducted a questionnaire survey of 534 college students to understand their sleep patterns and intestinal health problems. Twenty-two healthy volunteers were selected to explore the influence of SD and SR on gut microbiome composition and metabolites, mainly butyrate. Furthermore, we successfully established a 72-h sleep deprivation mouse model to explore the influence of SD on small intestinal mucosal damage, to explore the intervention role of butyrate in it, and expand our knowledge about the mechanisms by which butyrate acts in lipopolysaccharide (LPS)-treated intestinal epithelium cells (IECs).

## RESULTS

### Questionnaire survey.

With regard to sleep pattern and intestinal health, we conducted a questionnaire survey among 534 college students. A total of 30 questions were included (see Table S1 in the supplemental material), and among them, a detailed analysis of 8 questions was performed (Fig. S1). Interestingly, 85.39% of the students suffered from severe sleep deficiency, with less than 7 h of sleep per day (Fig. S1A). Moreover, 70.79% of the students went to bed after 23:00 every day (Fig. S1B), and 17% of students had a sleep latency of more than 30 min (Fig. S1C). Moreover, almost 72.85% of the students displayed frequent insomnia (Fig. S1D). Therefore, 39.33% of the students still felt tired and lethargic, and only 12.36% of the students felt energetic (Fig. S1E). Furthermore, insufficient sleep also led to decreased work and study efficiency and changes in emotions, including drowsiness (74.34%), anxiety and irritability (57.68%), decreased learning efficiency (53.37%), memory loss (51.12%), decreased physical resistance (35.02%), acne (32.77%), etc. (Fig. S1F). In addition, all 534 students also suffered from various gastrointestinal problems, such as indigestion (41.76%), bloating (13.86%), belching (12.17%), abdominal cramps (11.99%), heavy stomach (11.80%), and abdominal pain (7.68%) (Fig. S1G). Even worse, while 31 of 534 students were diagnosed with IBD, the remaining 503 students may still have undiagnosed bowel disorders (Fig. S1H). These data revealed that the prevalence of IBD was as high as 5.81%, which requires special attention.

Overall, among the 85.39% of students who slept less than 7 h, 41.76% of students suffered from various bowel disorders, suggesting a high correlation between insufficient sleep and intestinal diseases.

### Experimental setup and sleep analysis in SD and SR volunteers.

**FIG 1:**
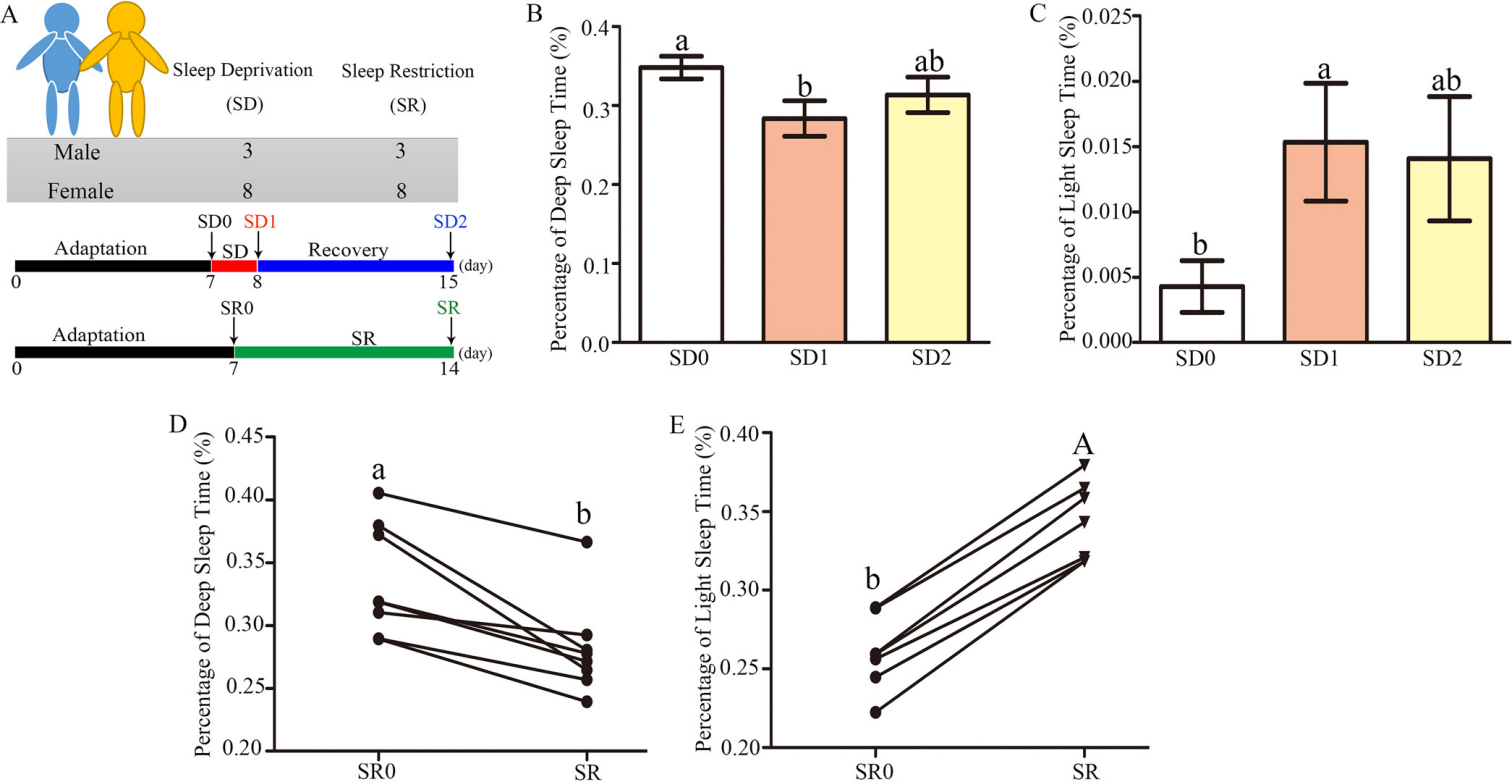
Experimental setup and sleep analysis in SD and SR volunteers. (A) Illustration of the experimental design. Twenty-two healthy college students participated in SD or SR experiments (each with 11 volunteers [3 males and 8 females]). All volunteers had a 7-day adaptation period. Two different sleep study experiments were set: acute SD (24 h in total) and chronic SR (less than 7 h in 7 nights instead of 8 h in bed per night) after the end of the 7-day adaptation period. SD began at 08:00 on the 8th day (SD0) and ended at 08:00 on the 9th day for SD1, without sleep supplementation during the day until evening, restoring the schedule with an adaptation period and with a recovery period of 7 days for SD2. In the second protocol, subjects received a 7-day adaptation period (SR0) followed by seven nights of SR of 5 h per night (02:00 to 07:00), ending on the 14th day (SR). The *x* axis represents “day.” (B and C) Duration of deep sleep (B) and duration of light sleep (C) in the SD0, SD1, and SD2 groups. (D and E) Duration of deep sleep (D) and duration of light sleep (E) in the SR0 and SR groups. Values are presented as mean ± SE. Differences were assessed by ANOVA and are denoted as follows: different lowercase letters indicate *P* < 0.05, different uppercase letters indicate *P* < 0.01, and the same letters indicate *P* > 0.05.

Figure 1A illustrates the experimental design for SD and SR experiments. In the SD group, the heart rates before going to bed and after waking up were significantly reduced by 20.1% (*P* = 0.000) (Fig. S1I) and 16.8% (*P* = 0.001) (Fig. S1J), respectively, compared with those in the SD0 group. However, the sleep heart rate and wake-up heart rate were increased by 22.4% (*P* = 0.000) and 17.2% (*P* = 0.015) (Fig. S1K and L), respectively, in the SR group versus the control SR0 group. SD and SR had similar effects on deep sleep and light sleep durations (Fig. 1B to E). In the SD and SR groups, deep sleep duration was significantly reduced by 18.6% (*P* = 0.026) (Fig. 1B) and 16.2% (*P* = 0.010) (Fig. 1D), while the light sleep duration was obviously upregulated by 238.6% (*P* = 0.044) (Fig. 1C) and 32.1% (*P* = 0.000) (Fig. 1E), respectively, compared with that in the SD0 and SR0 groups. However, after a 7-day recovery period, the deep sleep duration increased (10.6%) and the light sleep duration decreased (8.2%) in the SD2 group versus SD1 group, but none returned to the control SD0 level (Fig. 1B and C).

### Effects of SD and SR on gut microbiota α diversity and β diversity.

**FIG 2:**
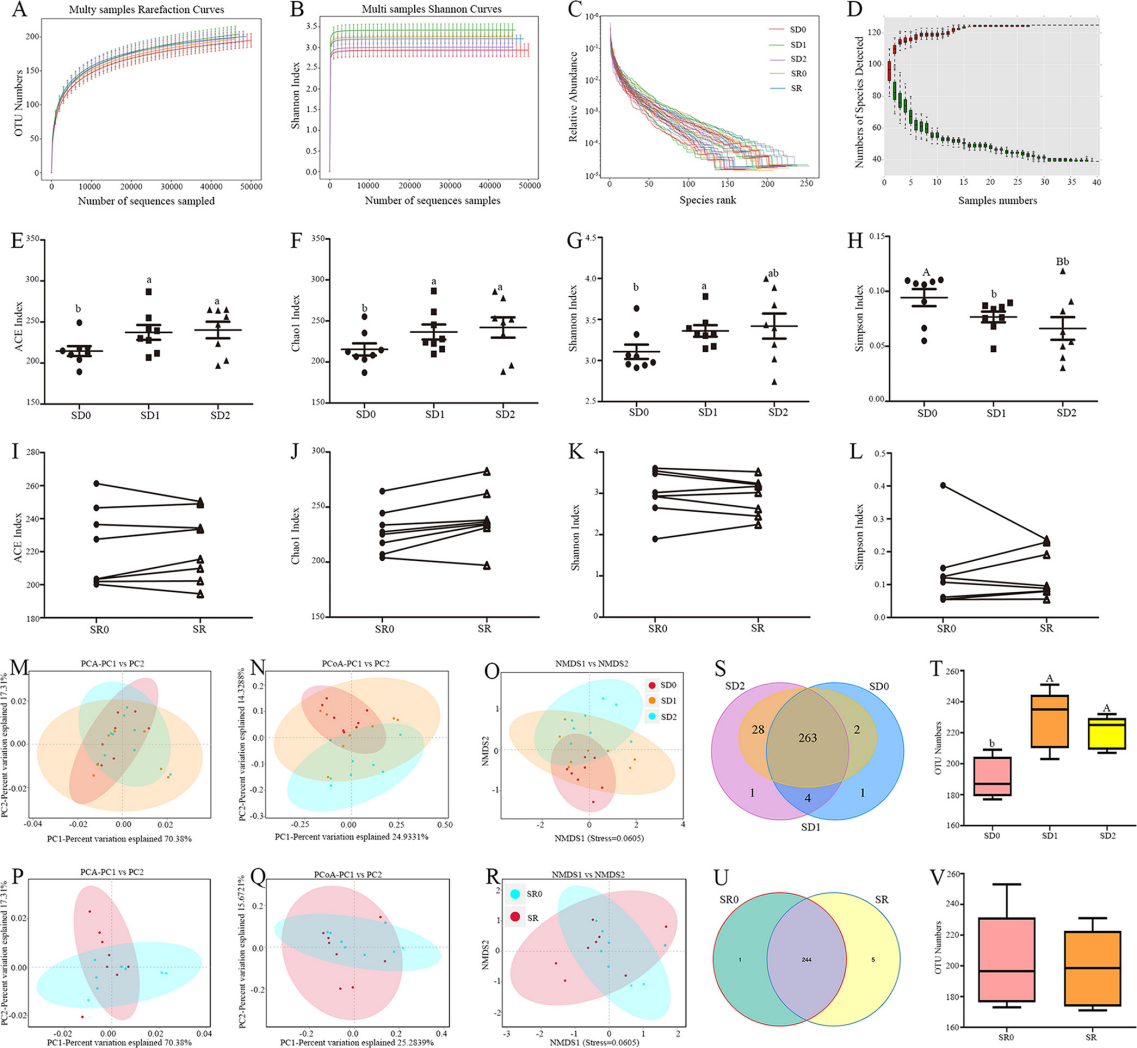
α diversity includes diversity and richness, and β diversity shows the degree of dispersion in the SD0, SD1, SD2, SR0, and SR groups. (A to D) OTU rank curves (A), Shannon curves (B), rarefaction curves (C), and the rank abundance curve (D); (E to H) ACE (E), Chao1 (F), Shannon (G), and Simpson (H) indices of the SD0, SD1, and SD2 groups; (I to L) ACE (I), Chao1 (J), Shannon (K), and Simpson (L) indices of the SR0 and SR groups; (M to R) PCA (M and P), PCoA (N and Q), and NMDS (O and R) score plots based on the Bray-Curtis score plot based on the OTUs for the SD0, SD1, SD2, SR0, and SR groups; (S to V) OTU numbers. (S and T) Venn diagram (S) and statistics (T) of OTU numbers for the SD0, SD1, and SD2 groups; (U and V) Venn diagram (U) and statistics (V) of OTU numbers for the SR0 and SR groups. Values are presented as mean 6 SE. Differences were assessed by ANOVA and are denoted as follows: different lowercase letters indicate *P* < 0.05, different uppercase letters indicate *P* < 0.01, and the same letters indicate *P* > 0.05.

Next, we investigated whether SD and SR induced alterations in intestinal microbiota composition. 16S rRNA high-throughput pyrosequencing suggested that the fecal microbiota produced 3,188,051 raw reads among the college students. After filtering of low-quality sequences, the 2,974,844 valid tags were assigned to various analyses (Table S2). The results of α-diversity analysis indicated the diversity and richness of fecal microbiota. In contrast, SR had no obvious influence on the richness and diversity of fecal microbiota (Fig. 2A to D). Quantitative perspective data showed the index of ACE, Chao1, and Shannon (Fig. 2E to G) were obviously upregulated by 12.0 to 13.8% (*P* = 0.017 to 0.049), 13.8 to 16.4% (*P* = 0.017 to 0.018), and 8.19 to 10.1% (*P* = 0.040 to 0.096), respectively, whereas the Simpson index (Fig. 2H) was obviously reduced by 21.0 to 31.9% (*P* = 0.000 to 0.011) in the SD1 and SD2 groups versus the SD0 group. However, there was no markedly significant diversity in these indexes between the SR0 and SR groups (*P* > 0.221) (Fig. 2I to L).

Moreover, β-diversity analysis of fecal microbiota composition showed distinct clustering in the SD and SR experiments (Fig. 2M to R). After SD, we observed an obvious downregulation in fecal microbiota aggregation, suggesting a reduction in fecal microbiota homogeneity, and aggregation failed to return to the control SD0 level after 7 days of recovery (Fig. 2M to O). Similarly, fecal microbiota dispersion upregulated in the SR group versus SR0 group (Fig. 2P to R). Statistically, the results (Fig. S2) indicated that the results for principal-component analysis principal component 1 (PCA-PC1) versus PC2, principal coordinate analysis principal-coordinate 1 (PCoA-PC1) versus PC2, and nonmetric multidimensional scaling 1 (NMDS1) versus NMDS2 increased by 24.5 to 34.5% (*P* = 0.010 to 0.025) (Fig. S2A to C) in the SD1 group compared with SD0 group, while there was a reduction (19.8 to 32.4%; *P* = 0.021 to 0.052) in the SD2 group relative to SD1 group. In SR group, the values of PCA-PC1 versus PC2, PCoA-PC1 versus PC2, and NMDS1 versus NMDS2 significantly increased by 31.2 to 42.6% (*P* = 0.010 to 0.030) (Fig. S2D to F) compared with the SR0 group.

All valid reads were clustered into operational taxonomic units (OTUs) based on a similarity level of 97%. Versus the SD0 group, there was a distinct upregulation in OTU number in the SD1 (19.9%; *P* = 0.001) and SD2 (15.5%; *P* = 0.008) groups (Fig. 2S and T). Yet, there was no obvious difference in OTU numbers between the SR and SR0 groups (Fig. 2U and V). Hence, our findings revealed that both SD and SR had the same effect on fecal microbiota dispersion; however, SD upregulated the OTU number, while SR had no effect on it.

### Effect of SD and SR on intestinal microbiota component.

**FIG 3:**
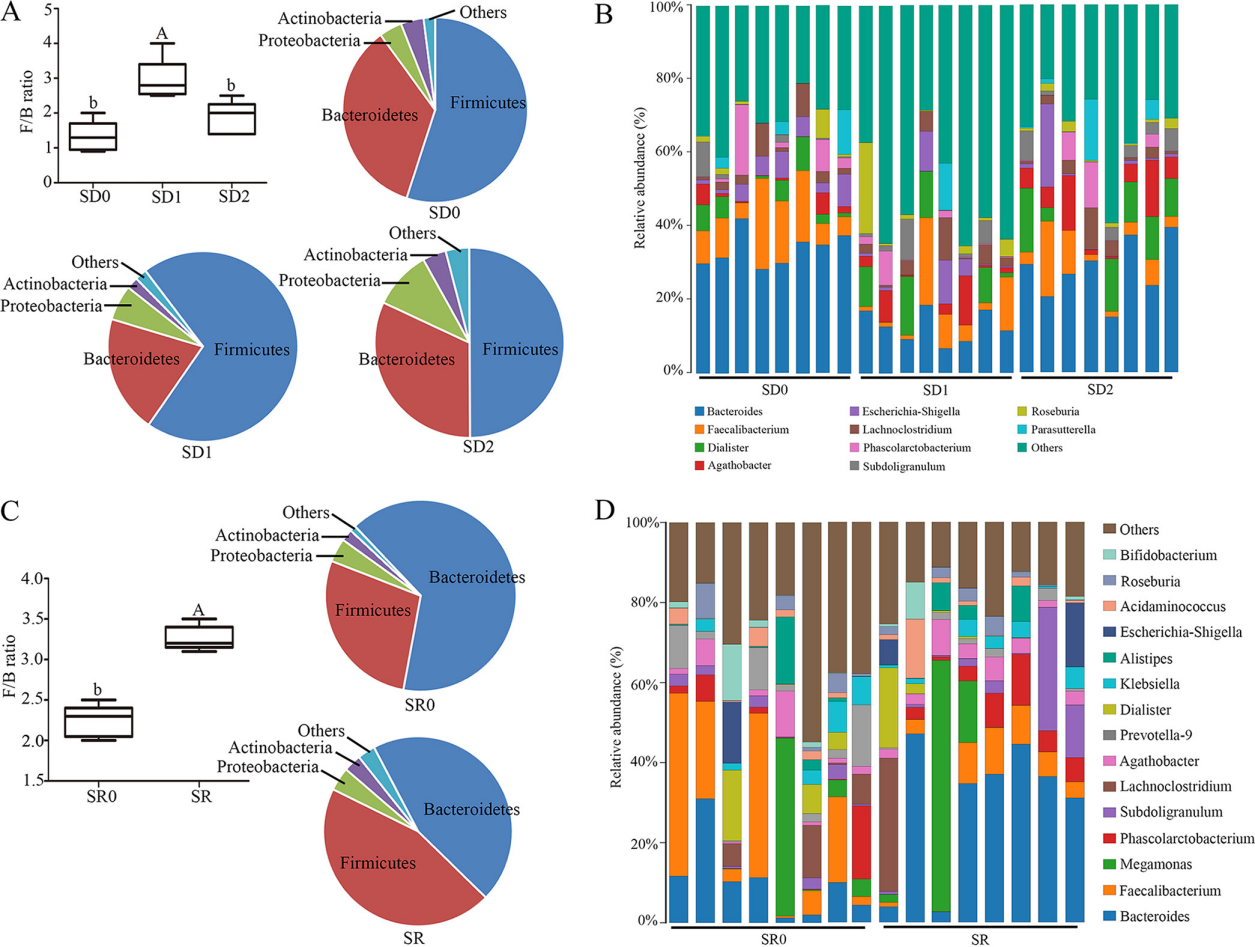
Composition of the fecal microbiotas in the SD0, SD1, SD2, SR0, and SR groups. (A and B) F/B ratio and relative contribution of the top 4 phyla (A) and relative abundance of the top 10 genera (B) in the SD0, SD1, and SD2 groups. (C and D) F/B ratio and relative contribution of the top 4 phyla (C) and relative abundance of the top 10 genera (D) in the SR0 and SR groups (D). Values are presented as mean ± SE. Differences were assessed by ANOVA and are denoted as follows: different lowercase letters indicate *P* < 0.05, different uppercase letters indicate *P* < 0.01, and the same letters indicate *P* > 0.05.

A histogram of species distribution can intuitively reflect changes in fecal microbiota composition. Specifically, phylum content statistics indicated that SD obviously reduced the content of *Bacteroidetes* and *Actinobacteria* and upregulated that of *Firmicutes* and *Proteobacteria*, while the 7-day recovery period restored the content of *Firmicutes* and *Bacteroidetes* (Fig. 3A). Moreover, at the genus level (Fig. 3B), there was a remarkable upregulation in the content of *Dialister* and *Agathobacter*, but there was also an obvious reduction in that of *Bacteroides* and *Faecalibacterium*; however, the relative abundance of *Bacteroides* and *Agathobacter* returned to the SD0 level after the 7-day recovery period. Phylum-level analysis showed similar results in the SR and SR0 groups (Fig. 3C). However, genus content statistics indicated that the content of *Faecalibacterium*, *Prevotalla-9*, *Acidaminococcus*, and *Bifidobacterium* significantly decreased, but the content of *Bacteroides*, *Megaonas*, *Subdoligranulum*, *Agathobacter*, *Dialister*, and Escherichia*-Shigella* upregulated in the SR versus SR0 group (Fig. 3D). Similarly, the ratio of *Firmicutes* to *Bacteroidetes* (F/B ratio) of fecal microbiota significantly decreased in the SD1 (110.8%; *P* = 0.000) and SR (45.5%; *P* = 0.000) groups versus the SD0 and SR0 groups, respectively, which resulted in fecal microbiota-related disorders (Fig. 3A and C).

**FIG 4:**
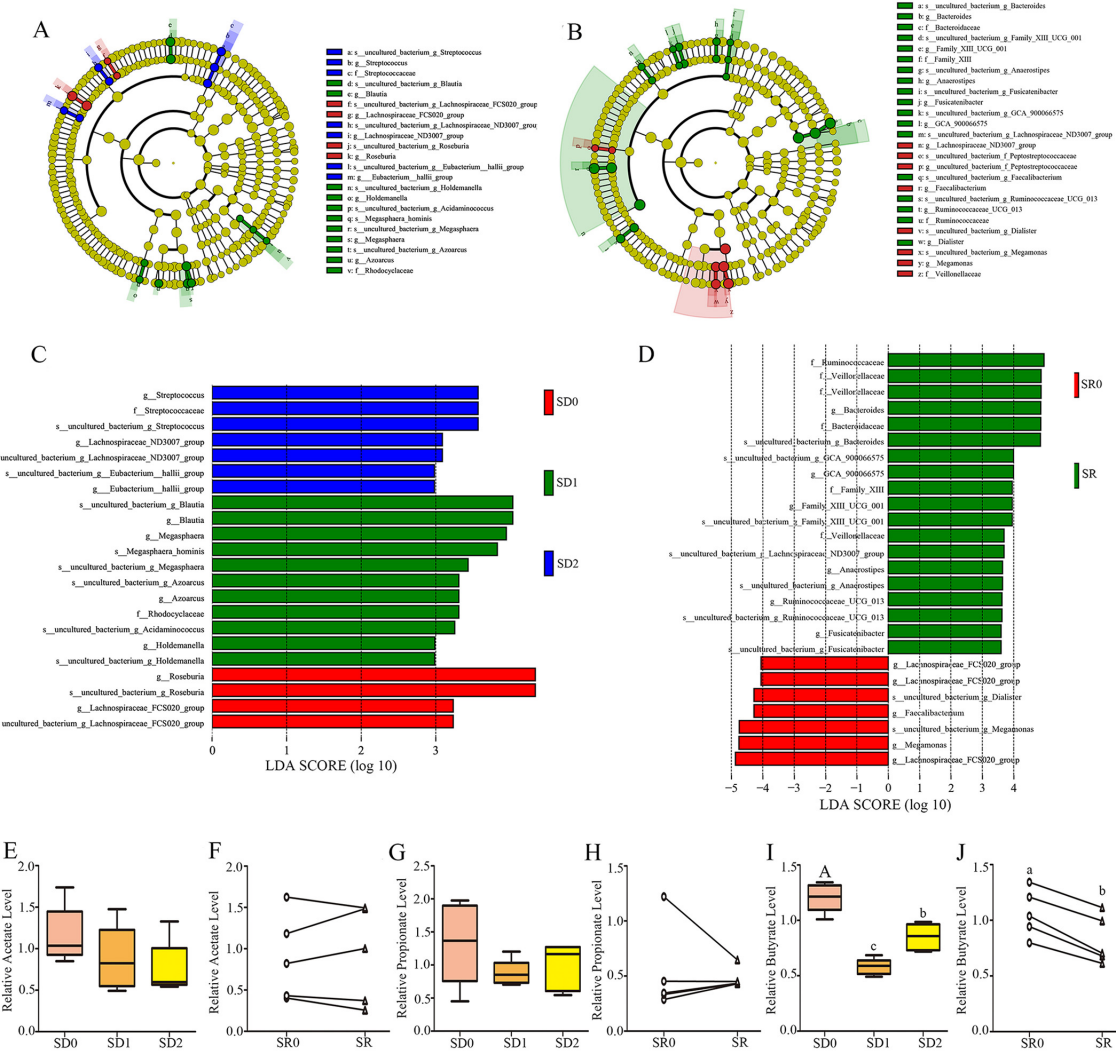
Dominant bacterial community in the fecal microbiotas of the SD0, SD1, SD2, SR0, and SR groups. (A and B) Taxonomic cladogram obtained from LEfSe (linear discriminant analysis effect size) sequence analysis in the SD0, SD1, and SD2 groups (A) and the SR0 and SR groups (B). Biomarker taxa are highlighted by colored circles and shaded areas. The diameter of each circle reflects the abundance of that taxa in the microbial community of the SD0, SD1, and SD2 groups (C) and the SR0 and SR groups (D). (E, G, and I) Relative acetate (E), propionate (G), and butyrate (I) levels in the SD0, SD1, and SD2 groups; (F, H, and J) relative acetate (F), propionate (H), and (J) butyrate (J) levels in the SR0 and SR groups. Taxa with differential abundance in the fecal microbiota of the SD0, SD1, SD2, SR0, and SR groups are indicated. A cutoff value of ≥2.0 was used for linear discriminant analysis (LDA). Values are presented as mean ± SE. Differences were assessed by ANOVA and are denoted as follows: different lowercase letters indicate *P* < 0.05, different uppercase letters indicate *P* < 0.01, and the same letters indicate *P* > 0.05.

Moreover, a cladogram representative of the fecal microbiota composition showed the key bacteria and the most notable difference in taxa between the SD and SR communities (Fig. 4). The predominant bacteria in the feces were *Lachnospiraceae* and *Roseburia* in the SD0 group, *Holdemanella*, *Megasphaera*, *Azoarcus*, *Rhodocyclaceae*, and *Blautia* in the SD1 group, and Streptococcus, *Lachnospiraceae*, and *Eubacterium* in the SD2 group. Most of the bacteria in the SD0 and SD2 groups were butyrate-producing bacteria, including *Lachnospiraceae* and *Roseburia* (Fig. 4A and C). Moreover, the predominant bacteria in the SR0 group were *Megamonas*, *Faecalibacterium*, and *Lachnospiraceae*, which are probiotics, and those in the SR group were *Anaerostipes*, *Fusicatenibacter*, and *Veillonellaceae*, which are pathogenic bacteria (Fig. 4B and D).

Furthermore, we found that SD and SR had no influence on the contents of acetate and propionate (Fig. 4E to H). However, the butyrate content was reduced by 53.1% (*P* = 0.000) in the SD1 group (Fig. 4I) and 30.7% (*P* = 0.014) in the SR group (Fig. 4J) compared with the SD0 and SR0 groups, respectively. However, after a 7-day recovery period, the butyrate content was increased by 46.5% in the SD2 group versus the SD1 group, and it failed to return to the control SD0 level (*P* = 0.002).

### Correlation analysis of gut microbiota.

There was a total of 80 significantly changed intestinal microbiotas among the three SD groups, and these changed 80 intestinal microbiotas interacted with each other to mediate intestinal function (Fig. S3A and B). Specifically, the abundances of *Bacteroides*, *Lachnospiraceae*, and *Faecalibacterium* were positively related to each other in the SD groups. Notably, the abundance of these three bacterial genera was significantly reduced in the SD1 group versus SD0 group, suggesting that they may play an improvement role in gut function. In the SR group, the abundance of *Bacteroides* was positively related to that of *Phascolarctobacterium* and *Faecalibacterium*. In addition, the abundance of *Phascolarctobacterium* also positively correlated with that of *Bacteroides*, while negatively correlated with that of *Prevotella-7* and *Holdemanella*. Meanwhile, the abundance of *Prevotella-9* was positively correlated with that of *Akkermansia* and negatively correlated with that of *Lachnospiraceae* and *Fusicatenibacter*. Among these bacteria, the abundance of *Faecalibacterium*, the main butyrate-producing bacteria, significantly decreased after SD and SR, suggesting that butyrate exerts an obvious beneficial effect on insufficient-sleep-induced bowel disease.

### Butyrate intervention ameliorated intestinal mucosa injury in sleep-deprived mice.

**FIG 5:**
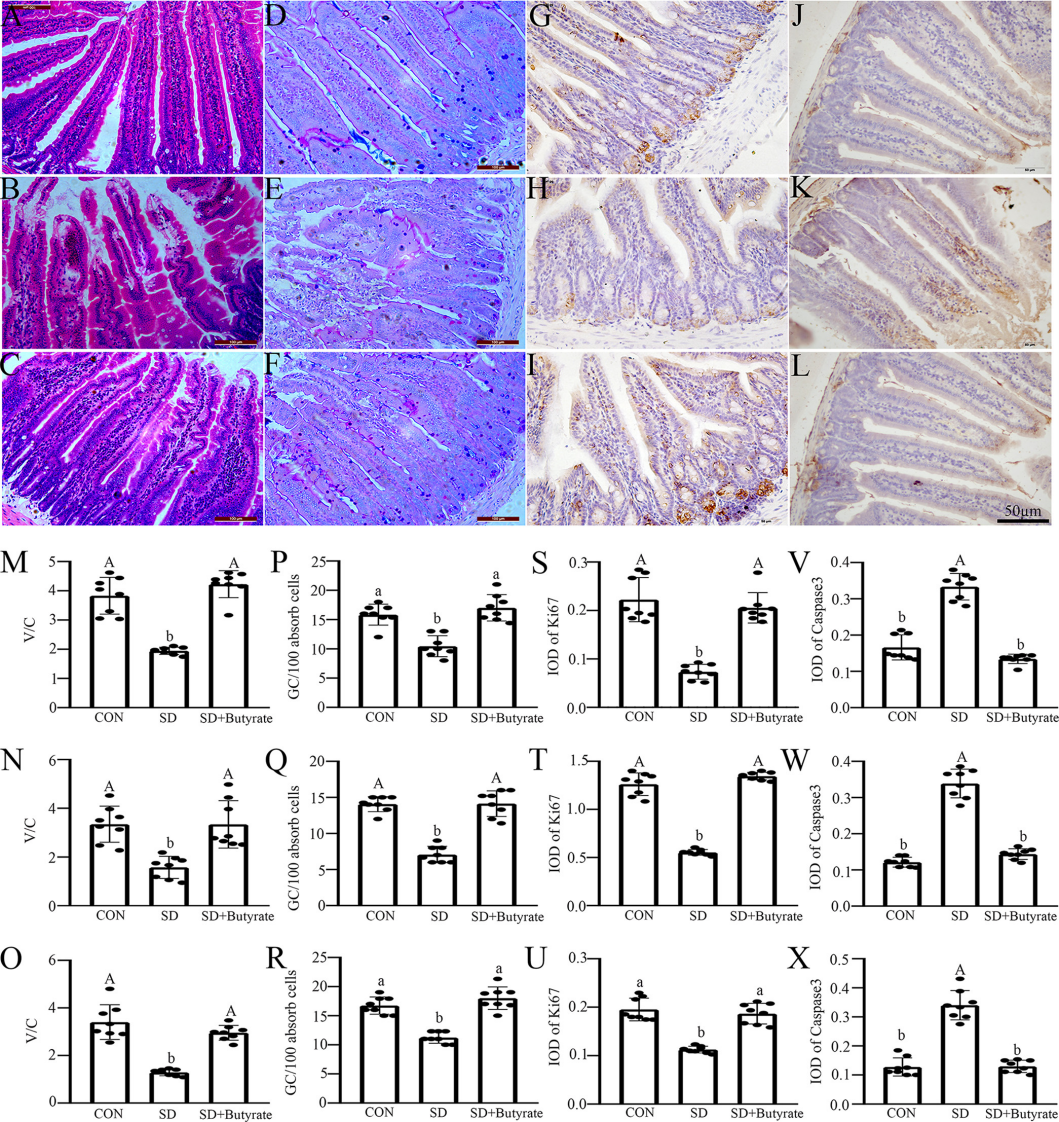
Effects of butyrate supplementation on the sleep deprivation-induced small intestines structure damage in mice. (A to L) HE staining (A to C), PAS staining (D toF), immunohistochemical staining of Ki67 (G to I) and caspase 3 (J to L) in duodenum of the control (CON), SD, and SD+Butyrate groups, respectively (scale, 50 μm). The V/C ratio (M to O), number of goblet cells per 100 absorbed cells (P to R), and IODs of Ki67 (S to U), and caspase 3 (V to X) were measured in duodenum (M, P, S, and V), jejunum (N, Q, T, and W), and ileum (O, R, U, and X), respectively. Values are presented as mean ± SE. Differences were assessed by ANOVA and are denoted as follows: different lowercase letters indicate *P* < 0.05, different uppercase letters indicate *P* < 0.01, and the same letters indicate *P* > 0.05.

**FIG 6:**
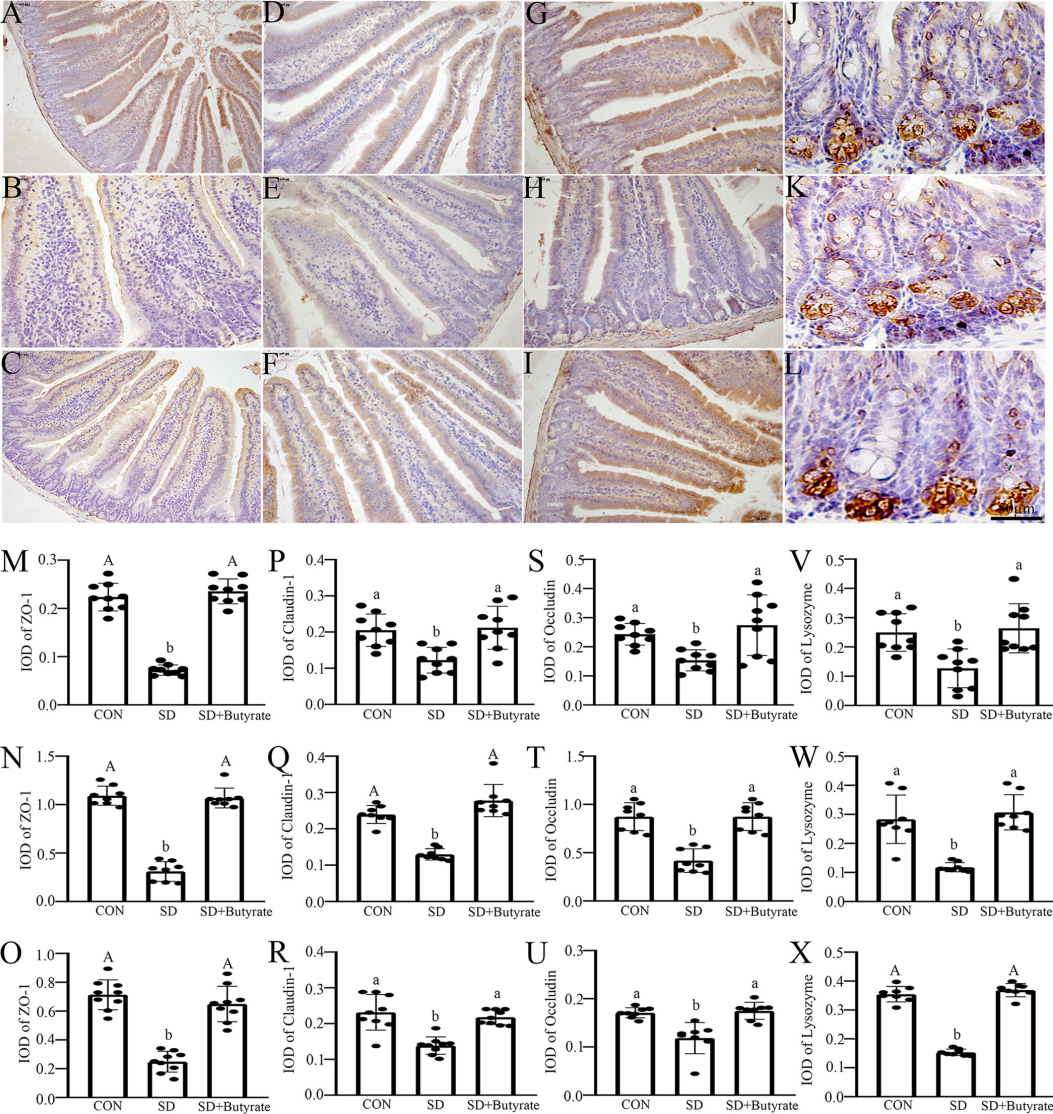
Effects of butyrate supplementation on the sleep deprivation-induced small intestinal mucosal injury in mice. (A to L) Immunohistochemical staining of ZO-1 (A to C), claudin-1 (D to F), occludin (G to I) and lysozyme (J to L) in duodenum of the control (CON), SD, and SD+Butyrate groups, respectively (scale bar, 50 μm). IODs of ZO-1 (M to O), claudin-1 (P to R), occludin (S to U), and lysozyme (V to X) were measured in duodenum (M, P, S, and V), jejunum (N, Q, T, and W) and ileum (O, R, U, X). Values are presented as mean ± SE. Differences were assessed by ANOVA and are denoted as follows: different lowercase letters indicate *P* < 0.05, different uppercase letters indicate *P* < 0.01, and the same letters indicate *P* > 0.05.

A 72-h sleep-deprived mouse model with or without butyrate intervention was used to reveal the positive regulatory effect of butyrate on the small intestines dysfunction caused by SD. The results of α-diversity analysis showed that the richness and diversity of fecal microbiota significantly decreased in the sleep-deprived mice compared with those in the control (CON) group, while butyrate supplementation could suppress the process. Quantitative perspective analysis showed that the ACE, Chao1, and Shannon indices were significantly decreased by 21.8 to 26.5% (*P* = 0.000 to 0.040) (Fig. S4A to C), while the Simpson index was markedly increased by 21.0% (*P* = 0.021) (Fig. S4D) in the SD group compared with the CON group. However, butyrate supplementation could restore these processes and result in no significant difference between the butyrate-treated group and the CON group (*P* > 0.050). Moreover, The hematoxylin and eosin (H&E) staining results (Fig. 5) indicated that the intestinal tissues of the sleep-deprived mice had significant fractures, especially at the top and bottom of the intestine’s villi (Fig. 5A to C). Statistically, the ratios of villus height to crypt depth (V/C ratios) were obviously reduced by 22.7 to 58.5% (*P* = 0.001 to 0.007) in duodenum, jejunum, and ileum, respectively, in SD group (Fig. 5M to O) compared with the control group. After butyrate supplementation, the V/C ratio was upregulated by 28.2 to 48.7% (*P* = 0.000) in small intestines related to the SD group. Moreover, periodic acid-Schiff (PAS) staining indicated that goblet cells were red, distributed between the intestinal villi and intestinal epithelial cells, and mostly located in the lower half of the villi (Fig. 5D to F). Statistically, compared with the CON group, the number of goblet cells per 100 absorbed cells was obviously reduced by 32.9 to 50% (*P* = 0.000 to 0.021) in duodenum, jejunum, and ileum of the sleep-deprived mice (Fig. 5P to R), whereas, butyrate supplementation suppressed the process, the number of goblet cells was upregulated by 35.4 to 50.2% (*P* = 0.000 to 0.027) in small intestines versus the SD group, and there was no obvious difference between the group supplied butyrate and the CON group (*P* > 0.072). These results revealed that butyrate could markedly ameliorate the intestinal digestion and absorption capacity and mucosal injury induced by SD. Furthermore, immunohistochemical staining indicated that SD attenuated the proliferative capacity but increased the level of apoptosis in the small intestines (Fig. 5G to I and J to L). Specifically, the integral optical densities (IODs) of Ki67 were reduced by 32.7 to 62.7% (*P* = 0.000 to 0.031) (Fig. 5S to U) in the duodenum, jejunum, and ileum, while there was an increased in the IOD of caspase 3 in the duodenum, jejunum, and ileum (52.8 to 65.1%; *P* = 0.000 to 0.001) (Fig. 5V to X) in sleep-deprived mice related to the control group, while butyrate supplementation restored the process. Moreover, SD altered the expression level of three tight junction proteins (Fig. 6). Specifically, there was a reduction in the IOD of ZO-1 (44.7 to 72.1%; *P* = 0.000) (Fig. 6A to C and M to O), claudin-1 (31.158.5%; *P* = 0.000 to 0.004) (Fig. 6D to F and 6P to R) and Occludin (54.2 to 58.3%; *P* = 0.000 to 0.110) (Fig. 6G to I and S to U) in small intestines of sleep-deprived mice versus the CON group. In contrast, butyrate intervention ameliorated the negative effect of SD on the changes in the distribution pattern of tight junction proteins in small intestines, and there was no obvious difference between the butyrate-supplied group and the control group (*P* > 0.137). Similarly, the results for western blotting suggested the same trend (Fig. S5).

We further explored whether SD and butyrate intervention could exert an improvement effect on the small intestinal mucosal immune barrier (Fig. 6 and Fig. S6). The immunohistochemistry consequence suggested the level of lysozyme (Fig. 6J to L and Fig. 6V to X), secretory IgA (S-IgA) (Fig. S6A to C and M to O), CD4^+^ (Fig. S6D to F and P to R), CD8^+^ T cells (Fig. S6G to I and S6S to U), and defensin-3 (Fig. S6J to L and 6V to X) were decreased by 28.1 to 45.5% (*P* = 0.000 to 0.015) in duodenum, 32 to 52% (*P* = 0.000 to 0.012) in jejunum, and 30.1 to 56.5% (*P* = 0.000 to 0.026) in ileum in the sleep-deprived mice compared with the control mice. However, butyrate intervention suppressed the process, and there was no statistical significance between the butyrate-supplied mice and the control mice (*P* > 0.162).

### Butyrate intervention ameliorated inflammation response in sleep-deprived mice.

Moreover, our results indicated that SD induced a decrease of anti-inflammatory factors (Fig. S7), such as the levels of interleukin-10 (IL-10) and gamma interferon (IFN-γ), by 26.2 to 46.8% (*P* = 0.000 to 0.012) (Fig. S7A, E, and I) and 27.2 to 49.1% (*P* = 0.000 to 0.035) (Fig. S7B, F, and J) and an upregulation of proinflammatory factors, such as the levels of IL-6 and tumor necrosis factor alpha (TNF-α), by 34.7 to 58.7% (*P* = 0.000 to 0.025) (Fig. S7C, G, and K) and 35.4 to 62.5% (*P* = 0.000) (Fig. S7D, H, and L) relative to the CON group. Similarity, significantly increased levels of TNF-α were also detected in the stool of sleep-deprived people (SD and SR) compared with the control group (Fig. S8). However, after butyrate supplementation, the anti-inflammatory factors IL-10 and IFN-γ were upregulated by 32.5 to 72.8% (*P* = 0.000 to 0.012), whereas the levels of the proinflammatory cytokines IL-6 and TNF-α were reduced by 27.2 to 54.1% (*P* = 0.000 to 0.023) in the SD plus butyrate (SD+Butyrate) group relative to the sleep-deprived mice. Therefore, there was no obvious difference between the butyrate-supplied group and the CON group (*P* > 0.125).

### Butyrate supplementation inhibited oxidative stress in sleep-deprived mice.

**FIG 7:**
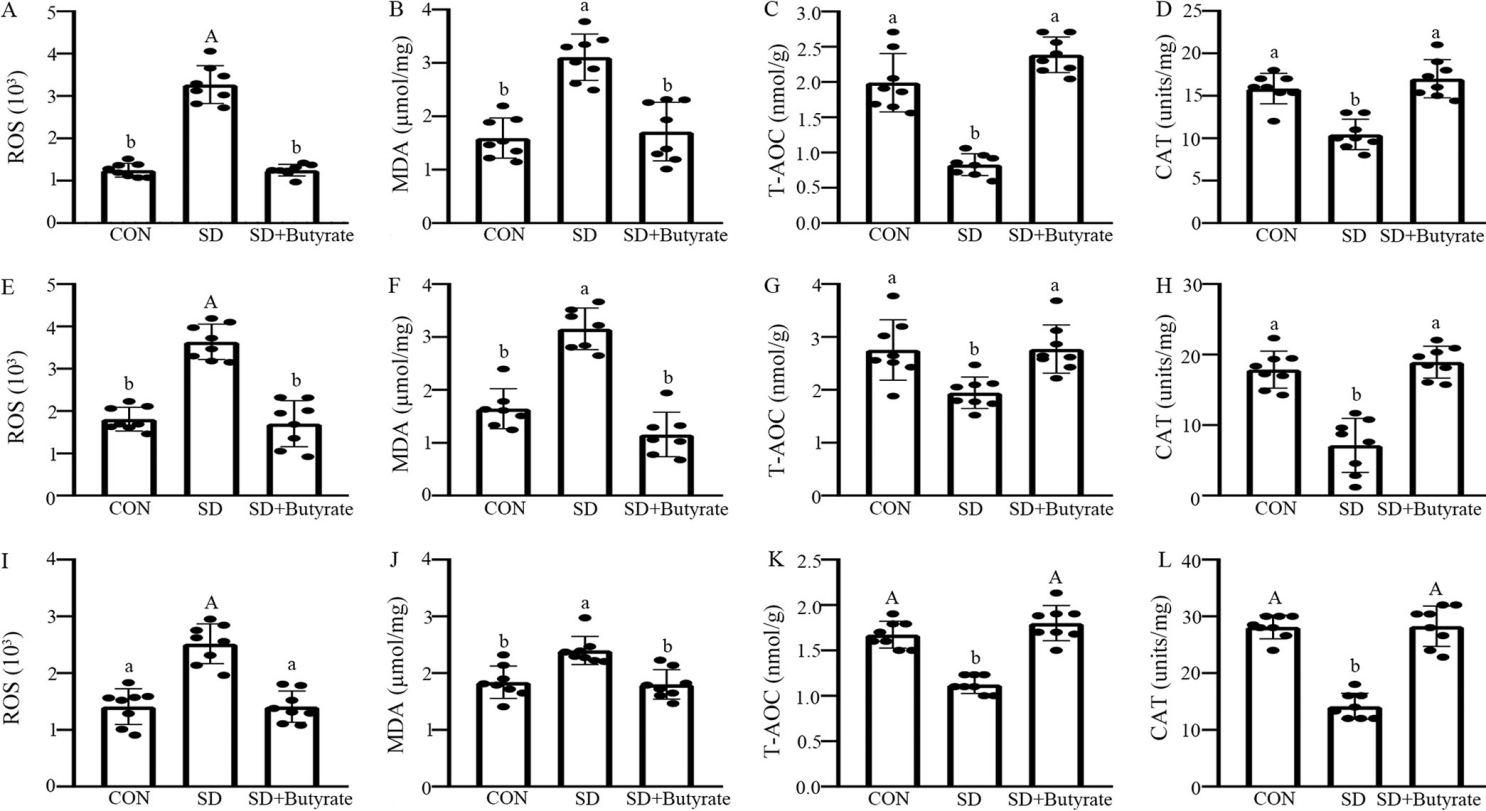
Effects of butyrate supplementation on the sleep deprivation-induced small intestines oxidative stress in mice. The contents of ROS (A, E, and I), MDA (B, F, and J), T-AOC (C, G, and K), and CAT (D, H, L) were measured in duodenum (A to D), jejunum (E to H), and ileum (I to L) of the CON, SD, and SD+Butyrate groups using an oxidative stress-related enzyme test, respectively. Values are presented as mean ± SE. Differences were assessed by ANOVA and are denoted as follows: different lowercase letters indicate *P* < 0.05, different uppercase letters indicate *P* < 0.01, and the same letters indicate *P* > 0.05.

Reactive oxygen species (ROS), antioxidant enzyme catalase (CAT), total antioxidant capacity (T-AOC), and malondialdehyde (MDA), were detected in small intestines (Fig. 7). The contents of ROS and the end product of lipid peroxidation MDA, were obviously increased by 32.0 to 47.8% (*P* = 0.000 to 0.018) in duodenum (Fig. 7A and B), 25.8 to 45.1% (*P* = 0.000 to 0.014) in jejunum (Fig. 7E and F), and 21.6 to 44.7% (*P* = 0.000 to 0.011) in ileum (Fig. 7I and J) of the SD group compared with the CON group. Conversely, the T-AOC and CAT contents were significantly reduced by 25.2 to 42.9% (*P* = 0.012 to 0.048) in duodenum (Fig. 7C and D), 19.7 to 32.5% (*P* = 0.024 to 0.045) in jejunum (Fig. 7G and H), and 21.3 to 43.4% (*P* = 0.000) in ileum (Fig. 7K and L) in the sleep-deprived mice compared with control mice. In contrast, after butyrate intervention, the negative effects of SD on the contents of ROS, MDA, T-AOC, and CAT were suppressed, resulting in no obvious difference between the butyrate-supplied group and the control group (*P* > 0.250). Furthermore, in SD mice, histone deacetylase 3 (HDAC3) and p-P65 proteins upregulated by 24.3 to 44.9% (*P* = 0.000 to 0.012) in duodenum (Fig. S9A and E), 32.7 to 44.8% (*P* = 0.000 to 0.033) in jejunum (Fig. S9G and K), and 46.5 to 55.2% (*P* = 0.000) in ileum (Fig. S9M and Q), while phospho-glycogen synthase kinase 3β (p-GSK-3β), β-catenin, Nrf2, and cyclin D1 proteins decreased by 32.9 to 85.3% (*P* = 0.000 to 0.012) in duodenum (Fig. S9B to D and F), 19.9 to 32.8% (*P* = 0.025 to 0.043) in jejunum (Fig. S9H to J and L), and 34.8 to 71.2% (*P* = 0.000 to 0.011) in ileum (Fig. S9N to P and R) in the SD group compared with the CON group, whereas, after butyrate intervention, the expression levels of HDAC3 and p-P65 proteins were reduced by 38.1 to 68.2% (*P* = 0.000 to 0.041) and the contents of p-GSK-3β, β-catenin, Nrf2, and cyclin D1 proteins were increased by 30.3 to 58.3% (*P* = 0.000 to 0.026) in the SD+Butyrate group, resulting in no obvious significance between the butyrate-supplied group and the control group (*P* > 0.576) (Fig. S9).

### Butyrate exerted an improvement effect through suppression of the HDAC3–GSK-3β–Nrf2–NF-κB loop.

**FIG 8:**
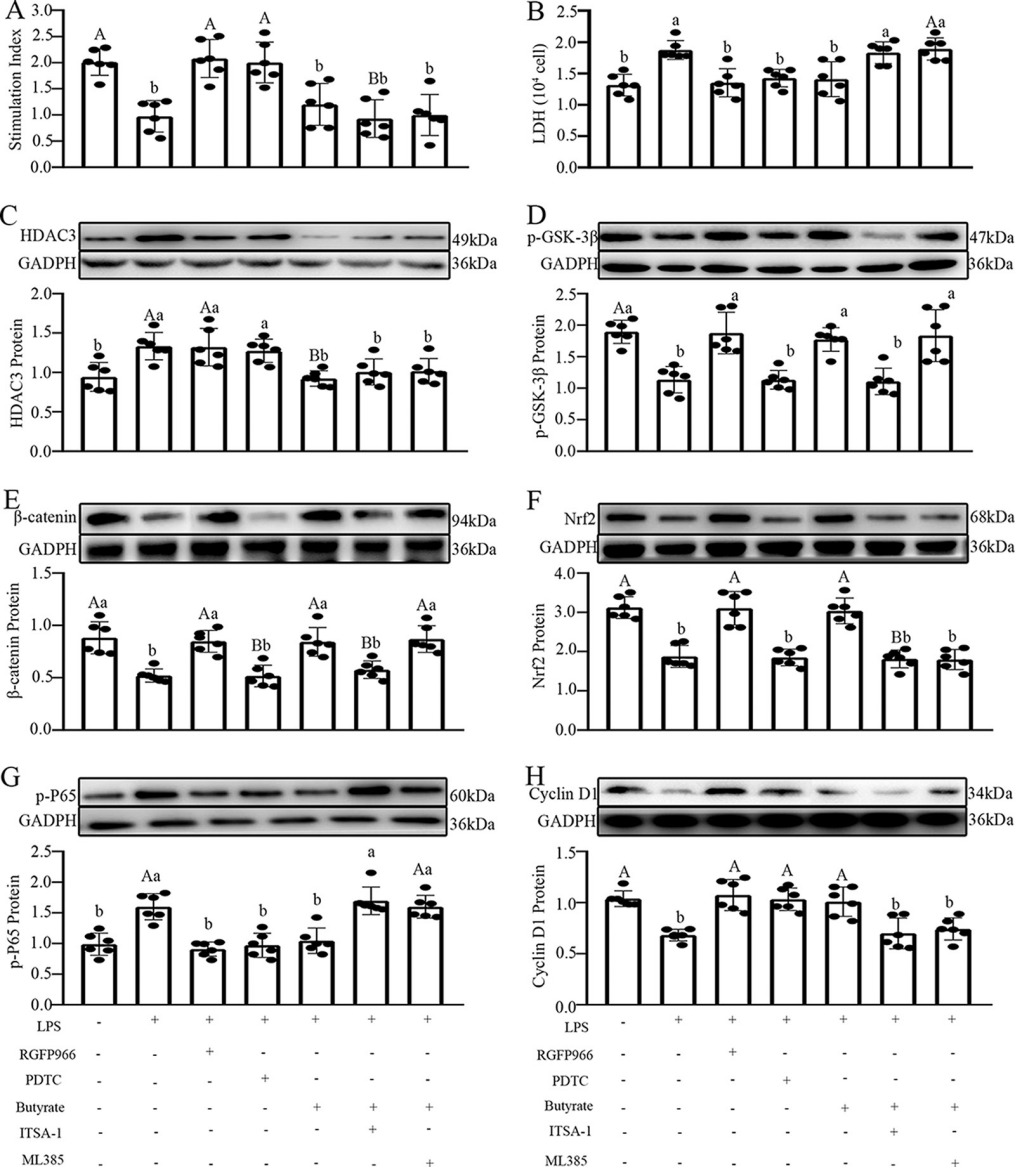
Effects of butyrate supplementation on the expression levels of signaling proteins in LPS-treated IECs. The stimulation index (A), LDH activity (B), and HDAC3 (C), p-GSK-3β (D), β-catenin (E), Nrf2 (F), p-P65 (G), and cyclin-D1 (H) protein expression in various treatment groups were examined by western blotting, and relative protein levels were normalized to glyceraldehyde-3-phosphate dehydrogenase (GAPDH). PDTC is an antagonist of NF-κB, ML385 is an antagonist of Nrf2, ITSA-1 is an agonist of HDAC3, and RGFP966 is an antagonist of HDAC3. Values are presented as mean ± SE. Differences were assessed by ANOVA and are denoted as follows: different lowercase letters indicate *P* < 0.05, different uppercase letters indicate *P* < 0.01, and the same letters indicate *P* > 0.05.

The feasible mechanism by which butyrate ameliorates SD- and SR-caused intestinal dysfunction was explored in an LPS-treated IECs (Fig. 8). Date indicated an obvious reduction in the cell proliferation index (17.6%; *P* = 0.004) (Fig. 8A) and the expression level of cyclin D1 protein (50.9%; *P* = 0.002) (Fig. 8H), as well as an increase in the lactate dehydrogenase (LDH) index (38.1%; *P* = 0.010) (Fig. 8B) in the LPS-treated group versus the vehicle group. Yet, butyrate supplementation significantly restored these LPS-caused negative effects. Furthermore, there were an upregulation of HDAC3 (59.2%; *P* = 0.008) (Fig. 8C) and p-P65 (57.2%; *P* = 0.000) (Fig. 8G) and a reduction of p-GSK-3β (36.7%; *P* = 0.000) (Fig. 8D), β-catenin (95.7%; *P* = 0.008) (Fig. 8E), and Nrf2 (42.3%; *P* = 0.000) (Fig. 8F) in the LPS group versus the CON group, whereas, butyrate pretreatment could suppress these processes. In contrast, after treatment with RGFP966 (an HDAC3 antagonist), which mimics the effect of butyrate, the results indicated a reduction of p-P65 (52.9%; *P* = 0.000) and the LDH index (35.4%, *P* = 0.025) and an upregulation of p-GSK-3β, β-catenin, Nrf2, cyclin D1, and cell proliferation (26.7 to 38.9%; *P* = 0.000 to 0.007) versus the LPS group. Treatment with ITSA-1, the agonist of HDAC3, offset the ameliorative effects of butyrate and failed to suppress the effects caused by LPS, indicating that butyrate ameliorated the LPS-induced inflammatory response by inhibiting HDAC3. Similarly, versus the control group, treatment with the Nrf2 antagonist ML385 induced an increase of p-P65 (59.3%; *P* = 0.007) and the LDH index (39.9%; *P* = 0.002) and a downregulation of cyclin D1 (40.5%; *P* = 0.006) and cell proliferation (18.4%; *P* = 0.001), but it had no effect on the expression levels of HDAC3, p-GSK-3β, and β-catenin proteins (*P* > 0.156). Meanwhile, versus the LPS group, PDTC (an antagonist of P65) treatment, which suppresses the activation of the NF-κB pathway, resulted in an upregulation of cell proliferation (19.3%; *P* = 0.005) and cyclin D1 (32.5%; *P* = 0.001) and downregulation of the LDH index (39.9%; *P* = 0.020). This suggested that the inflammatory response of LPS-treated IECs was triggered via NF-κB pathway activation (*P* > 0.055), further confirming the core position of the Nrf2–NF-κB loop in LPS-treated IECs. Hence, butyrate exerted its improvement effects through the HDAC3-mediated GSK-3β–β-catenin–Nrf2–NF-κB pathway.

## DISCUSSION

Previous research revealed that SD caused gut microbiota disturbance-mediated colonic barrier dysfunction in mice (17). Moreover, accumulating data in humans showed that the intestinal microbiota composition is altered in patients with IBD (2). Thus, we further explored whether insufficient-sleep-mediated intestinal microbiota imbalance induces small intestines dysfunction in college students and mice.

Moreover, we found that SR had no effect on the richness and diversity and numbers of OTUs of intestinal microbiota, while SD significantly increased these indicators. Our results of SD experiments were contradictory with other studies, which demonstrated that there were no obviously differences in the numbers of OTUs of intestinal microbiota between the SD and control groups (18), which may be partly rely on the differences in animal species (rats versus human) and SD duration. Similarly, in keeping with our results from SR experiments, Benedict et al. recognized that SR (the cycle is 2 days and includes sleeping for 4 h and 15 min a day) did not cause apparent influence on the composition or richness of intestinal microbiota, as revealed in young normal-weight individuals (19). Moreover, SD and SR caused a significant rise in the F/B ratio, which is a vital pointer of structural compositions of the intestinal microbiota (20). In general, both SD and SR induced intestinal microbiota imbalance, including downregulation of beneficial bacteria, such as *Bacteroidetes* and *Faecalibacterium*, which are the main butyrate-producing bacteria and have a positive influence on the synthesis of mucus glycans as well as the production of goblet cells in the colonic epithelium (20). Similarly, after 40 h of continuous SD, most of the altered microbiota were related to the modifications of SCFAs, including g_*Allobaculum*, g_*Alloprevotella*, g_*Prevotella*, and g_*Elusimicrobium*, which were markedly reduced (21). Furthermore, butyrate content significantly decreased after SD and SR, but no effect on the contents of acetate and propionate was observed, indicating that the decrease abundance of butyrate may have an important effect on inadequate-sleep-induced intestinal diseases. Among the SCFAs, specifically butyrate regulates various cellular processes (22, 23), which has been shown to have an influence on improving the inflammation response and stabilizing the colonic defense barrier (24–26). We further verified the beneficial effect of butyrate on insufficient sleep-induced intestinal mucosal damage and explored the mechanism of butyrate in intestinal diseases.

In our study, the results indicated that sleep deprivation disrupted the intestinal mucosal structure and function in mice. Specifically, there was a decrease in the ratio of V/C ratio and the number of goblet cells in the small intestines of sleep-deprived mice. Furthermore, the functional experiment demonstrated that the level of expression of tight junction proteins was significantly reduced in the small intestines as induced by sleep deprivation. Similarly, many researchers believe that the V/C ratio is a vital indicator to measure intestinal barrier function (27) and intestinal digestion and absorption capacity (28) of the small intestines. The mucus layer, synthesized and secreted by goblet cells, is another crucial defensive barrier in the gut: it has high molecular weight and highly glycosylated proteins that form a gel in the intestinal lumen (29). Furthermore, modifications of the distribution and number of tight junction proteins have an importantly influence on the permeability and integrity of the intestinal barrier (30). Furthermore, the lysozyme secreted by Paneth cells, S-IgA, and CD4^+^ and CD8^+^ T cells constitute an important immune barrier in the small intestines, and their numbers were significantly reduced after SD. Intestinal mucosal barrier damage in mice caused by SD results in a reduction in the proliferative activity of intestinal epithelial cells and an upregulation in the level of apoptosis. However, butyrate intervention plays an obviously ameliorative role on the SD-caused small intestinal mucosal damage. As an intestinal microbiota metabolite, researches suggested butyrate exerts an important influence on the intestinal homeostasis, maintaining homeostasis by protecting the integrity of intestinal mucosa barrier (31, 32), accelerating S-IgA synthesis (33), and adjusting T-cell proliferation and differentiation (10, 34). These results suggested that butyrate intervention significantly promoted the SD-caused relative intestinal mucosa barrier damage as well as the decreases in intestinal digestion and absorption.

We further explored the possible mechanism of how butyrate ameliorates SD-caused small intestinal mucosal damage. In the current study, butyrate intervention in sleep-deprived mice restored the SD-caused oxidative stress and inflammation responses. Particularly, research shows butyrate has anti-inflammatory activity in intestines (13, 35). Furthermore, we established an LPS-treated inflammatory IECs model, with or without butyrate supplementation, to explore the influence of butyrate on inflammation and its underlying mechanism. Our results showed that pretreatment of LPS-treated IECs with butyrate significantly increased cell proliferation. However, this effect was reversed with butyrate treatment, which demonstrated that butyrate promoted epithelial cell proliferation (9, 10). Research demonstrated that butyrate acts as an HDAC3 antagonist (36): our results suggested that butyrate supplementation suppressed the increase of HDAC3 protein induced by SD, and we also demonstrated that butyrate and RGFP966 increased cell proliferation and improved cell survival in LPS-induced IECs. In contrast, treatment with ITSA-1, an agonist of HDAC3, counteracted the positive effect of butyrate, which is consistent with the well-established function of HDAC3 as a negative regulator of gene transcription. Similarly, macrophages differentiated in the presence of butyrate also show enhanced antimicrobial functions and these effects have been confirmed to be mediated by HDAC3 inhibition (37). Furthermore, we noticed that silencing of HDAC3 significantly promoted the expression levels of p-GSK-3β and cyclin D1 proteins (see Fig. S10 in the supplemental material) in LPS-treated IECs, confirming the function of HDAC activation in LPS-induced proliferation inhibition; moreover, pretreatment of butyrate under HDAC3 silencing did not further promote the expression levels of p-GSK-3β and cyclin D1 proteins, confirming HDAC3 is required for the beneficial effect of butyrate. A previous study suggested that butyrate acts as an anti-inflammatory substance, mainly through NF-κB pathway inactivation in human colonic epithelial cells (35, 38), which was supported by the increased level of expression of p-P65 protein in inflammatory IECs in our study. However, our results further suggested that NF-κB pathway activation is not directly mediated by the upregulation of HDAC3. Results indicated that RGFP966 treatment promoted the expression of p-GSK-3β and β-catenin, further preventing NF-κB activation, while ITSA-1 treatment had the opposite effect. Moreover, research has demonstrated that the GSK-3β–Nrf2–NF-κB signaling loop exerts a vital effect on LPS-induced inflammatory response. Zhu demonstrated that LPS caused serious lung damage in mice via regulating the GSK-3β–Nrf2 signaling loop (39). Moreover, research also indicated that LPS induced BV2 microglial cells via activation of the GSK-3β–Nrf2 signaling loop (40). Here, we demonstrated that downregulation of p-GSK-3β and β-catenin inhibited IECs proliferation by activating the NF-κB pathway. Similarly, present evidence has also revealed that GSK-3β could improve cancer via regulating the NF-κB signaling pathway cascade via promoting the transcriptional activity of NF-κB in the nucleus (41). In addition, increased levels of ROS in inflammatory IECs indicated the occurrence of oxidative stress, which is mediated by the downregulation of Nrf2. Nrf2 inhibits oxidative stress by promoting the expression of antioxidant proteins (42–44). Furthermore, we found that treatment with ML385, an Nrf2 antagonist, had no effect on the level of p-GSK-3β protein and did not inhibit downstream NF-κB pathway activation. Mechanically, Nrf2 is anchored by GSK-3β, while oxidative stress induces GSK-3β activation and consecutive reduction of Nrf2-mediated antioxidant gene expression, thereby limiting cellular antioxidant capacity (45, 46). Therefore, we speculated that the HDAC3–p-GSK-3β–Nrf2–NF-κB loop exerts a vital beneficial effect on butyrate enhancing the decrease in intestinal cell proliferation in LPS-treated IECs.

In conclusion, our results revealed that sleep deficiency caused qualitative and selective changes in the gut microbiota composition and decreased the contents of *Faecalibacterium* and its metabolite butyrate, ultimately causing small intestinal mucosal injury. Furthermore, we demonstrated that pretreatment with butyrate effectively ameliorated small intestinal mucosal damage through the HDAC3–p-GSK-3β–Nrf2–NF-κB loop in mice and LPS-induced IECs (Fig. S11). Hence, the findings suggested that butyrate can be treated as a probiotic to ameliorate insufficient sleep-caused intestinal homeostasis imbalance and provide an advanced understanding of the mechanisms underlying the beneficial effect of butyrate.

## MATERIALS AND METHODS

### Survey design.

Questionnaires were randomly distributed among contemporary college students (see Table S1 in the supplemental material). The content of the questionnaire was mainly for the investigation of sleep and intestinal conditions. A total of 534 questionnaires, were collected and all were valid.

### Human subjects.

Subjects who met the following inclusion criteria were eligible for study participation: body mass index (BMI) below 30 kg/m^2^, appropriate physical and mental health as tested by physical detection and history, no clinically obvious abnormalities in blood chemistry; getting 8 to 9 h of sleep each day with habitual bedtimes during 22:00 to 23:00 and habitual awakenings at about 07:00 (recorded via wrist actigraphy and sleep logs throughout the test), no extreme morning and night habits, no disturbance in circadian rhythm, no mental illness, no severe stress from SD, no bad habits such as alcohol or drug use, and not currently under any medical or drug treatments as assessed by the questionnaire. The research agreements were allowed by the Institutional Review Board of the China Agricultural University (approval no. CAU2019014). Furthermore, all volunteers did not suffer from any intestinal disease and did not experience regular or intermittent intestinal discomfort. All volunteers eat normally, and there were no vegetarians. For every volunteer, written informed consent was obtained relying on the guidelines of the Declaration of Helsinki; all subjects received compensation for participation.

Twenty-two healthy college students, 20 to 25 years of age (mean age, 23.6 years; standard deviation, 2.01), participated in SD (11 volunteers [3 males and 8 females]) or SR (11 volunteers [3 males and 8 females]) experiments. All volunteers had a 7-day adaptation period between 8 and 9 h daily with habitual bedtimes between 22:00 and 23:00 and habitual awakenings at about 07:00. We set up two different sleep study experiments: acute SD (24 h in total) and chronic SR (less than 7 h in 7 nights instead of 8 h in bed per night) after the end of the 7-day adaptation period. SD began at 08:00 on the 8th day (SD0) and ended at 08:00 on the 9th day for SD1, without sleep supplementation during the day until evening, restoring the schedule with the adaptation period and with a recovery period of 7 days for SD2. In the second protocol, the volunteer accepted a 7-day adaptation period (SR0) followed by seven nights of SR of 5 h every night (02:00 to 07:00), ending on the 14th day (SR). The specific experimental method is provided in the supplemental material.

### Diet.

All participants indicated that they did not smoke, drink alcohol, drink coffee, or consume any medication during or prior to the experiment. Furthermore, all volunteers were prohibited from taking extra-high-calorie and high-energy foods. In order to avoid the influence of meal timing and food composition on intestinal microbiota composition, all volunteers' diets and meal timings were maintained the same. Furthermore, to explore the effect of sleep on gut microbiota composition, all volunteers were given a low-fat diet without sugar, which consisted of the following: breakfast included yogurt (Arla) (3 g fat/100 g) and natural cereal (ICA) (7 g fat/100 g) at 09:00, lunch included Bolognese (Findus) (2 g fat/100 g) at 13:00, and dinner included potatoes, beef, and vegetables with seed oil and onions (Findus) (5.5 g fat/100 g) at 20:00. Each meal had to be eaten within 20 min.

### Actigraphy.

Actigraphy was performed with a wrist watch (Actiwatch 2; Philips Respironics, Inc., Pittsburgh, PA) that was worn on the nondominant hand during the test for recording purposes. The collected data included total sleep duration, sleep time period, deep sleep duration (including time period), light sleep duration (including time period), wake up time, sleep heart rate, and wake-up heart rate, which were provided by the Actiwatch.

### Collection of stool samples.

Sterile stool collection tubes (catalog no. K708; WA Products) were distributed to volunteers for in-experiment stool collection prior to experimentation. Moreover, before the experiment, volunteers were trained on how to collect feces. Twenty-two healthy college students participated in SD (11 volunteers) or SR (11 volunteers) experiments. Therefore, 11 fecal samples could be collected in each of the 5 test baselines (SD0, SD1, SD2, SR0 and SR). Although the method of collecting feces was fully detailed and the volunteers were trained, there were still inevitable errors. Therefore, we selected 8 fecal samples that best met the collection criteria for analysis to objectively solve practical problems. Therefore, 40 samples (22 volunteers in two settings representing the baseline and experimental nodes) were used for follow-up analyses of microflora structure and SCFA level.

### Intestinal microbiota analysis.

Collected human fecal contents were stored at −80°C followed by bacterial genomic DNA extraction using a QIAamp DNA stool minikit from Qiagen (Hilden, Germany). The 16S rRNA gene comprising the V3-V4 regions was amplified via PCR using composite specific bacterial primers. The specific experimental procedure is provided in the supplemental material.

### SCFA extraction and analysis.

Collected human fecal contents were stored at −80°C. The stool sample was admixed with water and then centrifuged. The supernatant obtained after filtration was mixed with ether and sulfuric acid. After high-speed centrifugation, the ether layer was gathered and the SCFA concentration was detected using an Agilent 6890 N gas chromatograph.

### Animals and treatments.

A total of 36 male CD1 mice (8 weeks of age) (Vital River Laboratory Animal Technology Co., Ltd., Beijing, China) were used in this experiment. Each group of 6 mice were housed in one cage under normal circumstances (at a temperature of 21 ± 1°C and relative humidity of 50% ± 10%). The lighting system was on a 14-h light/10-h dark cycle (with lights on at 07:00.). All mice had free access to water and food. After a 1-week acclimation period, all mice were randomly divided into the following three groups: the control (CON) group, the sleep-deprived group (SD), and the sleep-deprived plus butyrate supplementation (SD+Butyrate) group. Continuous sleep deprivation of mice was initiated from 08:00 for 3 days via a modified multiple-platform water bath. The specific experimental method is provided in the supplemental material. For treatment with butyrate, 40 mM sodium butyrate (Sigma-Aldrich, St. Louis, MO, USA) was administered orally to mice by gavage (SD+ABs+Butyrate) 60 min before SD and at a fixed dose every day at 07:00 for 3 consecutive days.

After the experiment ended at 08:00, all mice were euthanized under anesthesia with 2% sodium pentobarbital (2.5 mL/kg, 0.1 mL per mouse). We collected mouse serum and three small intestines samples, including duodenum, jejunum, and ileum.

(Note that the previously published data in reference 47 have an important supporting role in the publication of this article and are very necessary for this article. In addition, the previously published data can well support the conclusions stated in this article and are conducive to the readers' understanding and better dissemination of the article.)

### ELISA.

The levels of inflammatory factors (TNF-α, IL-10, IL-6, and IFN-γ) in the small intestines of three groups in mice were determined by competitive enzyme-linked immunosorbent assay (ELISA) (Uscn Life Science, Inc., Wuhan, China). All experimental operations were carried out in accordance with the manufacturer’s instructions. There were eight biological replicates per group and three mechanical replicates per tissue. The intra-assay coefficient of variation (CV) was <10%, and the interassay CV was <12%. Data were measured on a microplate reader equipped with a 450-nm-pore filter. The levels of IL-6, TNF-α, IL-10 and IFN-γ concentration in the intestines were expressed as picograms per milligram of protein.

### Histological staining and PAS staining.

Small intestine tissue was fixed in 4% paraformaldehyde for 48 h and then embedded in paraffin and subsequently cut into 5-μm sections, which were stained with hematoxylin and eosin (H&E) or periodic acid-Schiff (PAS). At least 10 different fields of view were randomly taken per slice at ×400 magnification using a BX51 microscope (Olympus, Tokyo, Japan). The 5 longest villi in each field of view were selected for analysis, including villus height, crypt depth, ratio of villus length to crypt depth (V/C ratio), and the number of goblet cells per 100 epithelial cells from H&E staining and PAS staining. The V/C ratio represents the digestion and absorption capacity of the small intestines.

### Immunohistochemical staining.

For immunohistochemical staining, sections were incubated with monoclonal rabbit anti-mouse antibody overnight at 4°C (claudin-1, 1:200; occludin, 1:100; ZO-1, 1:200; Ki67, 1:500; caspase3, 1:200; lysozyme, 1:100; S-IgA, 1:100; CD4^+^, 1:100; CD8^+^, 1:100; defensin-3, 1:150) (Abcam, Cambridge, MA, USA). Sections were then rinsed with 0.01 M phosphate-buffered saline (PBS) (pH 7.4) and incubated with biotinylated goat anti-rabbit IgG (1:200) (Sigma, St. Louis, MO) at room temperature for 2 h. After rinsing, streptavidin-horseradish peroxidase (1:250) (Sigma, St. Louis, MO) was overlaid on the tissue and incubated for 2 h at room temperature. Tissues were overlaid with 0.01 M PBS containing 0.05% 3′,3-diaminobenzidine tetrahydrochloride (DAB; Sigma, St. Louis, MO) and 0.003% hydrogen peroxide and incubated in the dark for 10 min, followed by observation of immunoreactivity. Sections were subsequently stained with hematoxylin. In the experiment, a negative-control group with PBS without primary antibody should be set. Cells stained in yellowish brown are positive cells. Five fields of view were randomly selected for each section and the mean integral optical density (IOD) of the positive cells was counted. IOD represents the positive rate of positive cells: the larger the value, the more positive cells.

### Detection of oxidative stress and lipid peroxidation.

Small intestine tissue was instantly homogenized (200 × *g* for 10 min) and centrifuged at 4°C, after which the clarified supernatant was stored at −80°C and aspirated for subsequent manipulation. Tissue catalase (CAT) activity, total antioxidant capacity (T-AOC), and malondialdehyde (MDA) level were determined using colorimetric methods provided in commercial kits (Nanjing Jiancheng Co., Ltd.). There were eight biological replicates per group and three mechanical replicates per tissue.

### Western blotting.

Small intestine tissue and IECs samples were homogenized in liquid nitrogen and then stored at −80°C for later western blotting. The specific experimental procedure is described in the supplemental material.

### Determination of ROS formation.

The determination of ROS content was carried out according to the instructions of Sigma-Aldrich. After the small intestine tissues of each group were made into a single-cell suspension, the intracellular ROS content was tested by flow cytometry, and the oxidation-sensitive DCFH-DA fluorescent probe was used. Each biological replicate contained at least 19,105 cells, and at least 3 mechanical replicates were performed. The value was examined by a fluorescent microplate reader (with excitation 488 nm and emission 525 nm).

### Cell culture and treatment.

The human normal colon IECs (NCM 460) obtained from the RIKEN Cell Bank (Ibaraki, Japan). IECs were raised in Dulbecco's modified Eagle’s medium (DMEM) supplemented with 100 U/mL penicillin, 100 μg/mL streptomycin, and 10% fetal bovine serum, under the basic condition of 37°C and 5% CO_2_. The specific experimental procedure is described in the supplemental material.

### LDH assessment.

An LDH detection kit (Solarbio, Beijing, China) was used to detect LDH activity in cell supernatant, and all procedures were carried out according to the manufacturer’s instructions. LDH activity was measured at 450 nm via a microplate reader (model 680; Bio-Rad, St. Louis, MO, USA) and expressed as units per 10^4^ cells. There were eight biological replicates per group and three mechanical replicates per tissue.

### Statistical analysis.

The data are expressed as mean ± standard error (SE) and were analyzed using SPSS 10.0 software (SPSS, Inc., Chicago, IL, USA) with distribution tested before generation of data statistics. When comparing SD0, SD1, and SD2, we used a one-way repeated-measures analysis of variance (ANOVA). When comparing the SR0 and SR groups, we first performed a normality test on the data, and then performed a paired *t* test after it was satisfied (significance at *P* < 0.05 and *P* < 0.01).

### Data availability.

The sequence data have been deposited in the SRA archive (https://www.ncbi.nlm.nih.gov/sra) under accession no. PRJNA904026.
