# Supplementary material for: Butyrate Ameliorates Insufficient Sleep-Induced Intestinal Mucosal Damage in Humans and Mice
Source: Microbiol Spectr. 2022 Dec 21;11(1):e02000-22. doi: 10.1128/spectrum.02000-22 (PMC9927315; doi:10.1128/spectrum.02000-22)
Supplement: Supplemental file 1 — Supplemental material. Download spectrum.02000-22-s0001.pdf, PDF file, 1.7 MB [file spectrum.02000-22-s0001.pdf]

Supplementary Materials

Materials and Methods

Human Subjects

Every volunteer had to fill out the log after getting up and before going to sleep within 30 min every day, as well as record the heart rate, sleep quality and quantity on the night before, the time taken to fall asleep, the feeling after waking up, and the diet and exercise of the day. Throughout the study, dormitory conditions were highly controlled in terms of environmental conditions and scheduled activities. Ambient light was fixed at <50 lx during scheduled wakefulness, and <1 lx (darkness) during scheduled sleep periods. Ambient temperature was maintained between 22 and 24 °C. Subjects were restricted from exercising or engaging in strenuous activities, although they were allowed to read, play video or board games, watch television, and interact with laboratory staff to help remain awake (no visitors were permitted).

Gut Microbiota Analysis

Thermal cycling conditions were as follows: 95 °C for 5 min (1 cycle), 95 °C for 30 s, 50 °C for 30 s, 72 °C for 40 s (25 cycles), and a final extension at 72 °C for 7 min. High-throughput pyrosequencing of the PCR products was performed on an Illumina MiSeq platform (Biomarker Technologies Co, Ltd., Beijing, China). The raw paired-end reads from the original DNA fragments were merged using FLASH32 and assigned to each sample according to the unique barcodes. All effective reads from each sample were clustered into operational taxonomic units (OTUs) based on a 97% sequence similarity according to UCLUST. For α-diversity analysis, we rarified the OTUs to several metrics, including the curves of the OTU rank, rarefaction, and Shannon, and calculated the Shannon, Chao1, Simpson, and ACE indices. For β-diversity analysis, a heatmap of redundancy analysis (RDA)-identified key OTUs, principal component analysis

(PCA), principal coordinate analysis (PCoA), and nonmetric multidimensional scaling (NMDS) were performed using QIIME. LEfSe analysis was performed for the quantitative analysis of biomarkers among each group. Briefly, the LEfSe analysis, LDA threshold of > 4, used the nonparametric factorial Kruskal-Wallis (KW) sum-rank test and then the (unpaired) Wilcoxon rank-sum test to identify the most differently abundant taxa. The difference in dominant pathways between groups was detected using Kyoto Encyclopedia of Genes and Genomes (KEGG) analysis and clusters of orthologous groups of proteins (COG) difference analysis.

Animals and Treatments

Eighteen platforms were placed in a water tank. Twelve mice were placed in the water bath. Every mouse in the water bath could move from one platform to another by jumping. The water filled the water bath 4 cm from the base. When the mice had reached the rapid eye movement stage of sleep, which is the paradoxical phase of sleep, the mice will fall into the water caused by muscle atonia. The mice then woke and would try to climb up the platform to avoid being drowned. Throughout the experiments, the water was replaced with clean water in the tank.

Cell Culture and Treatment

After 6 h, the medium was changed to basal medium. After 12 h of cultivation, 5×10^6 cells/mL and 5×10^5 cells/mL were seeded per well in a flat-bottom 96-well culture plate and 12-well culture plate, respectively. Cell proliferation was assessed from the 96-well culture plates using a colorimetric assay based on the reduction of tetrazolium salt (3-(4,5-Dimethylthiazol-2-yl)-2,5-diphenyltetrazolium bromide (MTT, Sigma-Aldrich). The plates were incubated at 37 °C in 5% CO_2 for 24 h. Four hours later, 10 μL of MTT was added to the

medium (5 mg/mL in complete RPMI-1640 medium). At the end of 28 h, 100 μL of 10% sodium dodecyl sulfate (SDS) was added to each well. The optical density (OD) of each well was measured using a microplate reader (Model 680; Bio-Rad, St. Louis, MO, USA) equipped with a 570 nm wavelength filter. The mean OD values for each triplicate set were used in subsequent statistical analyses. The proliferative activity of IECs was expressed as the stimulation index (SI) as follows: SI = OD570 (stimulated cells)/OD570 (unstimulated cells).

In addition, some LPS (10 nM, Solarbio Ltd., Beijing, China)-treated cells from the 12-well culture plates were treated with 8 nM RGFP966 (a selective antagonist of the HDAC3; MCE, New Jersey, USA; LPS+RGFP966-cells), 100 μM PDTC (an NF-κB antagonist; MCE, New Jersey, USA; LPS+PDTC-cells), or 5 mM butyrate (Sigma-Aldrich, St. Louis, USA; LPS+Butyrate-cells). After butyrate supplementation for 30 min, some LPS+Butyrate-cells were sequentially treated with 50 μM ITSA-1 (a nonselective HDAC3 agonist; MCE, New Jersey, USA; LPS+Butyrate+ ITSA-1-cells) or 5 μM ML385 (a selective Nrf2 inverse antagonist; MCE, New Jersey, USA; LPS+Butyrate+ML385-cells). Importantly, the control group is the enzyme solvent group and the solvent is DMSO. Each plate of treated cells was incubated for 24 h and were collected for lactate dehydrogenase (LDH) assessment and western blotting. Each assay used a repeat of 8 wells.

Western Blotting

Total protein was extracted using lysis buffer (62.5 mmol/L Tris-HCl, 2% SDS, and 10% glycerol; pH 6.8). After centrifugation at 12,000 × *g* for 10 min at 4 °C, the supernatants were collected. Protein concentration was determined using a bicinchoninic acid (BCA) kit (Beyotime; P0012). A sample of 20 μg of protein was electrophoresed using 10% SDS-polyacrylamide gel

electrophoresis. After transferring the samples onto a PVDF membrane (Millipore, Billerica, MA, USA), p-P65 and p-GSK-3β proteins were blocked with 1% bovine serum albumin and other proteins were blocked with 5% skim milk in 1× Tris-buffered saline (TBS) with Tween (TBST) for 2 h at room temperature (23 °C ± 2 °C). Membranes were then incubated with monoclonal rabbit anti-mouse primary antibodies (GAPDH, 1:2000; p-P65, 1:1000; cyclin D1, 1:500; Nrf2, 1:1000; p-GSK-3β, 1:1000; β-catenin, 1:1000; HDAC3, 1:1000; Abcam, Cambridge, MA, USA) overnight at 4 °C. After washing with TBST, membranes were incubated with horseradish peroxidase-conjugated goat anti-rabbit IgG (1:5000, CW0103; CoWin Biotech Co., Inc.) for 2 h at 37 °C. Immunoblotting was performed using an ECL Western Blotting Kit (CW0049; CoWin Biotech Co., Inc.). The bands obtained in the blots were scanned and measured using ImageJ (version 4.0.2; Scion Corp., Frederick, MD, USA). Data are expressed as the integral optical density (IOD) of the bands. Experiments were repeated three times.

SiRNA Transfection

SiRNA oligonucleotides were designed and produced by Invitrogen (Grand Island, NY). Sequences (5`-3`) of HDAC3-targeting siRNA were CGGGAUGGCAUUGAUGACCAGAGUU (sense), AACUCUGGUCAUCAAUGCCAUCCCG (antisense); scramble RNA of High GC ratio was used as negative control. The siRNAs were transfected into cells using Lipofectamine RNAiMAX (Invitrogen) according to the manufacturer's instructions for 24 h before further operations, and the efficacy of silencing was assessed by immunoblotting.

Supplementary results

Figure S1

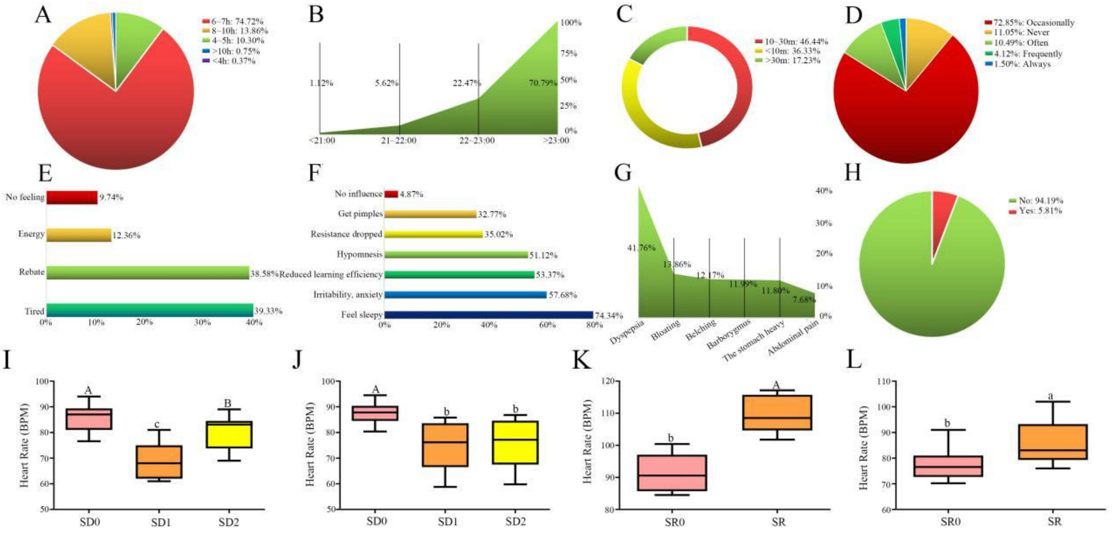

Questionnaire survey. Survey among 534 college students for (A) sleep duration, (B) bedtime, (C) fall asleep time, (D) insomnia, (E) wake up feeling, (F) effects of sleep insufficiency, (G) gastrointestinal problems, (H) and the proportion of patients with irritable bowel diseases. Values are presented as the mean ± SE. Differences were assessed by ANOVA and denoted as follows: different lowercase letters: $P < 0.05$; different uppercase letters: $P < 0.01$; same letter: $P > 0.05$.

Figure S2

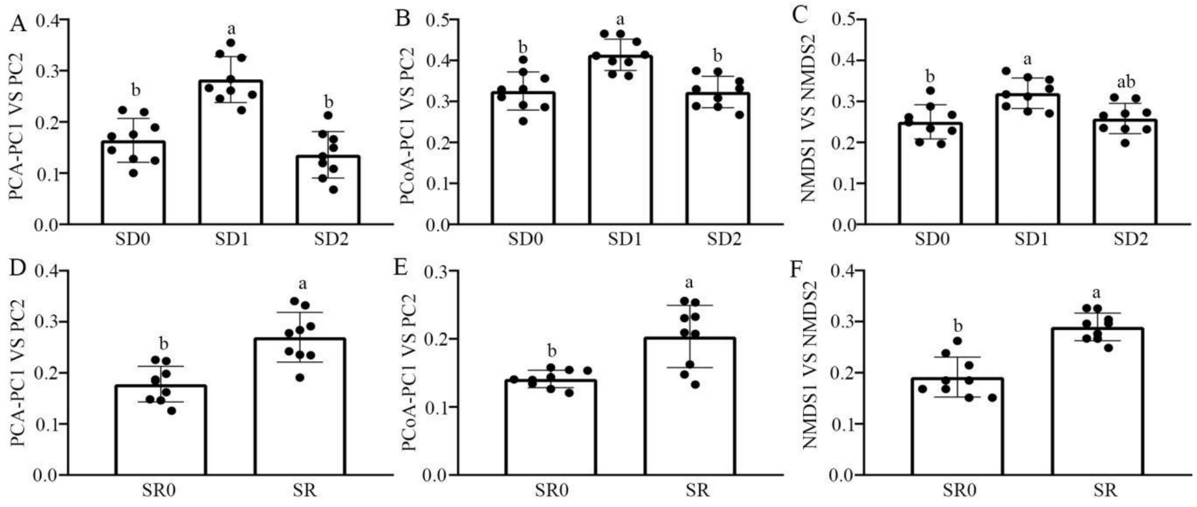

Effect of SD and SR on gut microbiota α diversity and β diversity. PCA (A, D), PCoA (B, E) and NMDS (C, F) score plots based on the Bray-Curtis score plot based on the OTUs for the SD0, SD1, SD2, SR0 and SR groups. Values are presented as the mean ± SE. Differences were assessed by ANOVA and denoted as follows: different lowercase letters: $P < 0.05$; different uppercase letters: $P < 0.01$; same letter: $P > 0.05$.

Figure S3

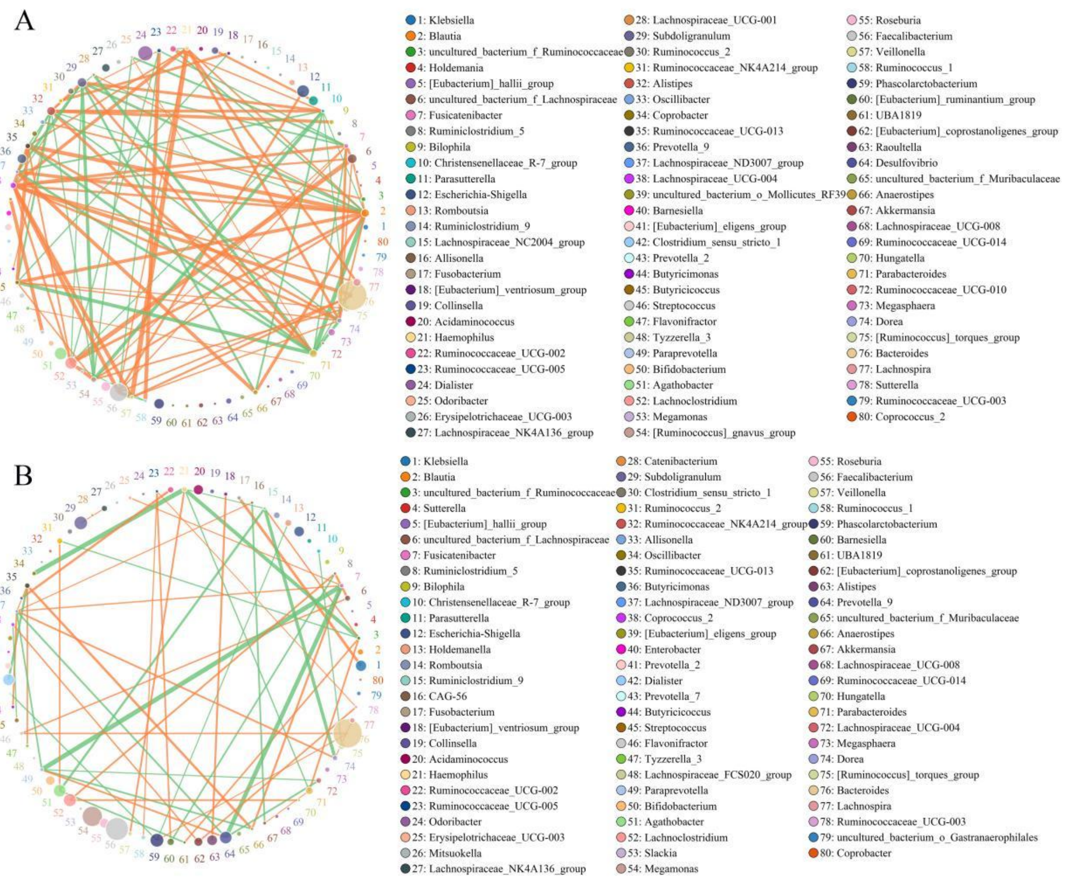

Correlation analysis of gut microbiota. 80 significantly changed intestinal microbiota among the three SD groups (A) and two SR groups (B), and these changed 80 intestinal microbiota interacted with each other to mediate intestinal function.

Figure S4

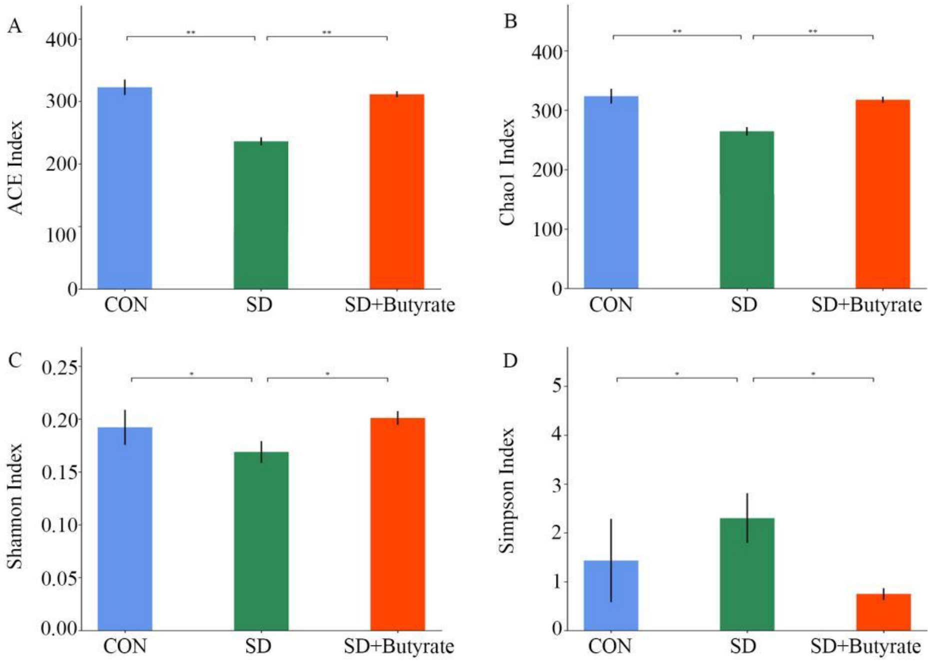

Effect of SD and butyrate supplementation on gut microbiota α diversity.

ACE (A), Chao1 (B), Shannon (C), and Simpson indices (D) of the CON, SD and SD+Butyrate groups. Values are presented as the mean ± SE. Differences were assessed by ANOVA and denoted as follows: different lowercase letters: $P < 0.05$; different uppercase letters: $P < 0.01$; same letter: $P > 0.05$.

Figure S5

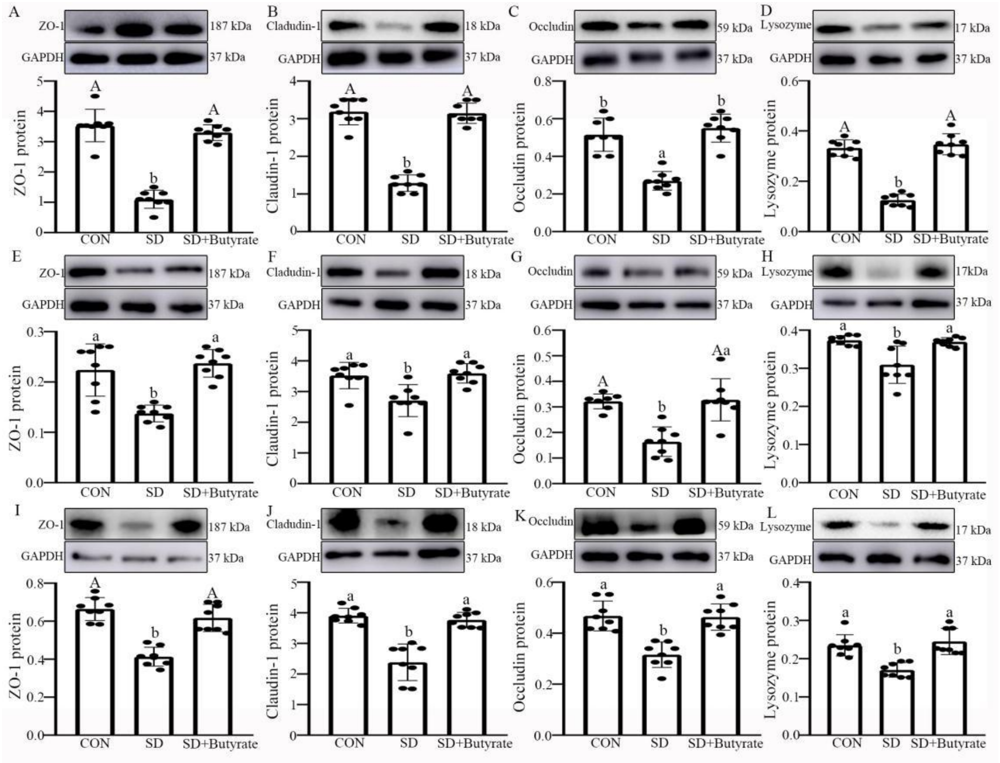

Butyrate intervention ameliorated the decrease of tight junction proteins (ZO-1, Claudin-1 and Occludin) in sleep deprived mice. The expression level of ZO-1 (A, E, I), Claudin-1 (B, F, J), Occludin (C, G, K) and Lysozyme (D, H, L) protein in duodenum (A-D), jejunum (E-H) and ileum (I-L) of CON, SD, SD + Butyrate groups was examined by western blotting, and relative protein levels were normalized to GAPDH. Values are presented as the mean ± SE. Differences were assessed by ANOVA and denoted as follows: different lowercase letters: $P < 0.05$; different uppercase letters: $P < 0.01$; same letter: $P > 0.05$.

Figure S6

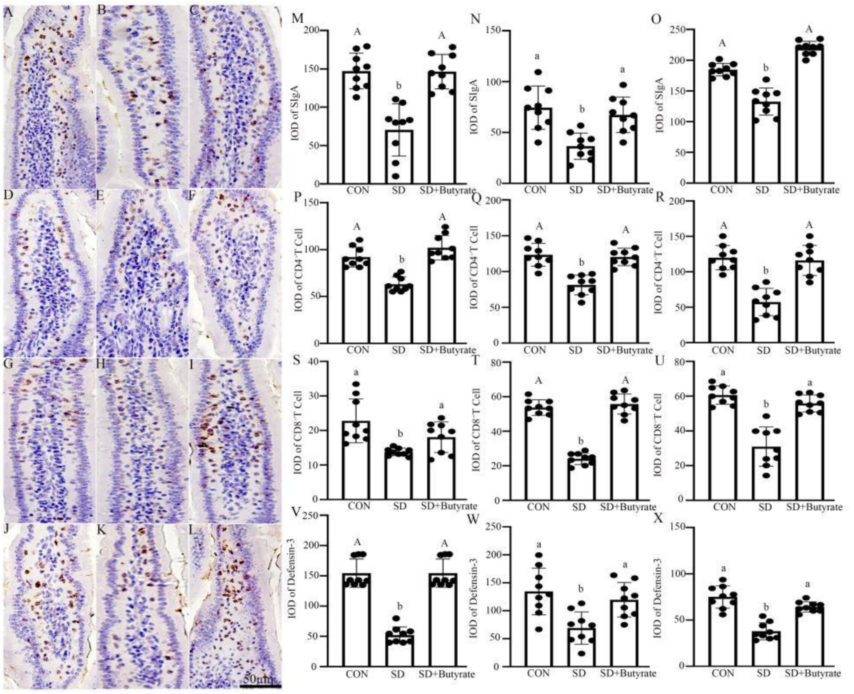

Butyrate intervention ameliorated the decreased immune function in sleep deprived mice. Immunohistochemical staining of SIgA (A-C), CD4^{+}T cell (D-F), CD8^{+}T cell (G-I) and Defensin-3 (J-L) in duodenum of CON, SD, SD + Butyrate groups, respectively (scale: 50 μm). IOD of SIgA (M-O), CD4^{+}T cell (P-R), CD8^{+}T cell (S-U) and Defensin-3 (V-X) were measured in duodenum (M, P, S, V), jejunum (N, Q, T, W) and ileum (O, R, U, X), respectively. Values are presented as the mean ± SE. Differences were assessed by ANOVA and denoted as follows: different lowercase letters: $P < 0.05$; different uppercase letters: $P < 0.01$; same letter: $P > 0.05$.

Figure S7

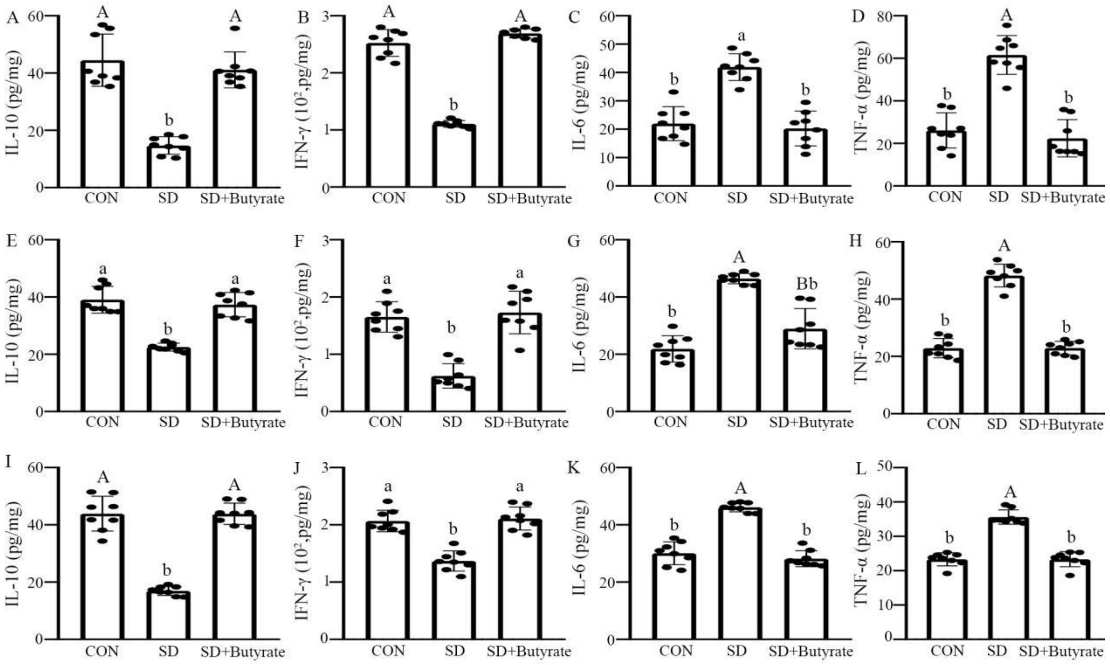

Butyrate intervention ameliorated inflammation response in sleep deprived mice. The content of IL-10 (A, E, I), IFN-γ (B, F, J), IL-6 (C, G, K) and TNF-α (D, H, L) were measured in duodenum (A-D), jejunum (E-H) and ileum (I-L) of CON, SD, SD + Butyrate groups using ELISA test, respectively. Values are presented as the mean ± SE. Differences were assessed by ANOVA and denoted as follows: different lowercase letters: $P < 0.05$; different uppercase letters: $P < 0.01$; same letter: $P > 0.05$.

Figure S8

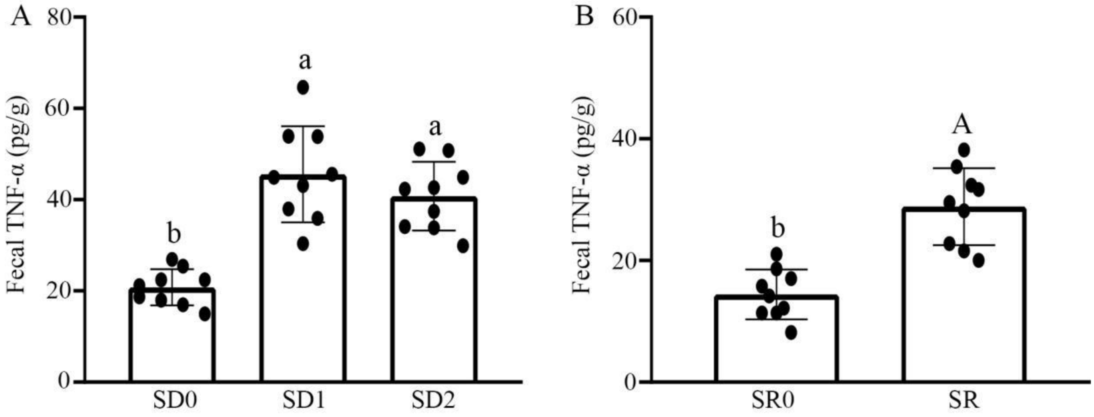

Sleep insufficiency induced inflammation response. The content of fecal TNF-α in SD0, SD1 and SD2 groups (A) and in SR0 and SR groups (B) using ELISA test. Values are presented as the mean ± SE. Differences were assessed by ANOVA and denoted as follows: different lowercase letters: $P < 0.05$; different uppercase letters: $P < 0.01$; same letter: $P > 0.05$.

Figure S9

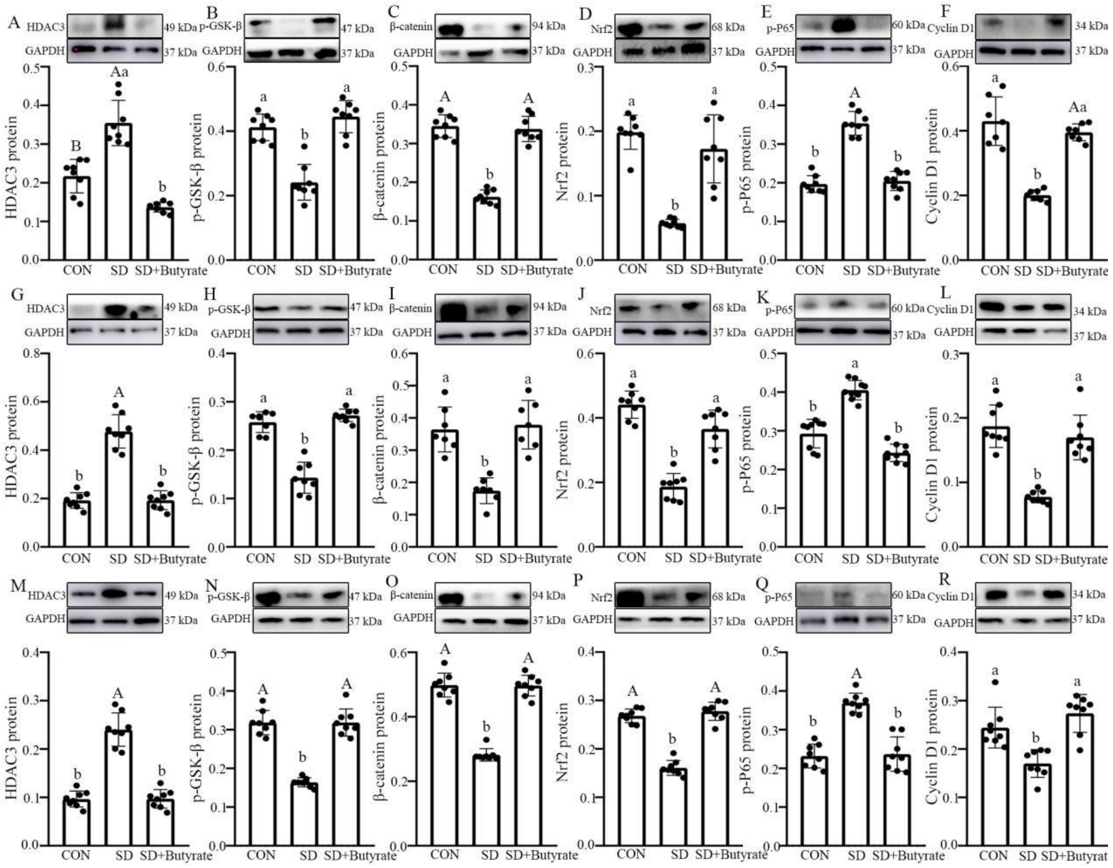

Butyrate supplementation improved the changes of pathway proteins expression in sleep-deprived mice. The expression level of HDAC3 (A, G, M), p-GSK-3β (B, H, N), β-catenin (C, I, O), Nrf2 (D, J, P), p-P65 (E, K, Q) and Cyclin D1 (F, L, R) protein in duodenum (A-F), jejunum (G-L) and ileum (M-R) of CON, SD, SD + Butyrate groups was examined by western blotting, and relative protein levels were normalized to GAPDH. Values are presented as the mean ± SE. Differences were assessed by ANOVA and denoted as follows: different lowercase letters: $P < 0.05$; different uppercase letters: $P < 0.01$; same letter: $P > 0.05$.

Figure S10

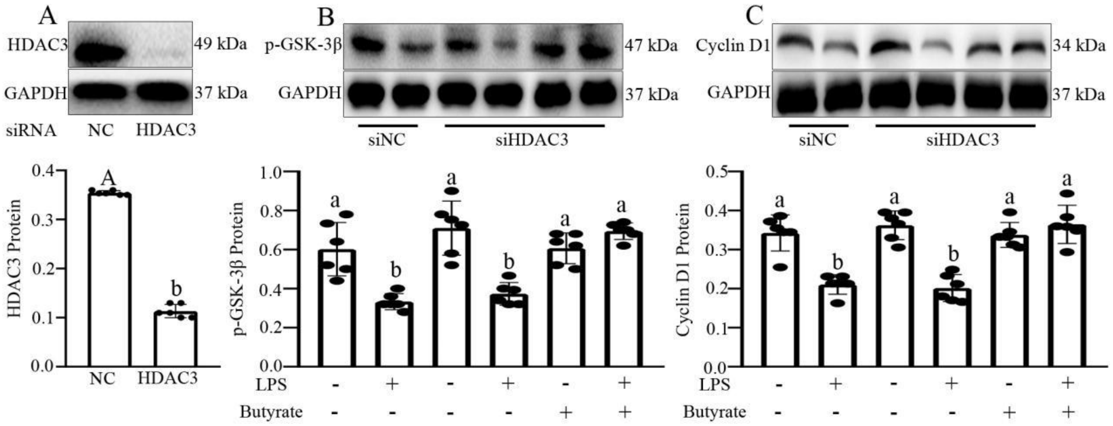

Butyrate-mediated HDAC3 inactivation improved LPS induced proliferative activity decreased. The expression level of HDAC3 (A), p-GSK-3β (B) and cyclin D1 (C) proteins in in various treatment groups was examined by western blotting, and relative protein levels were normalized to GAPDH. Values are presented as the mean ± SE. Differences were assessed by ANOVA and denoted as follows: different lowercase letters: $P < 0.05$; different uppercase letters: $P < 0.01$; same letter: $P > 0.05$.

Figure S11

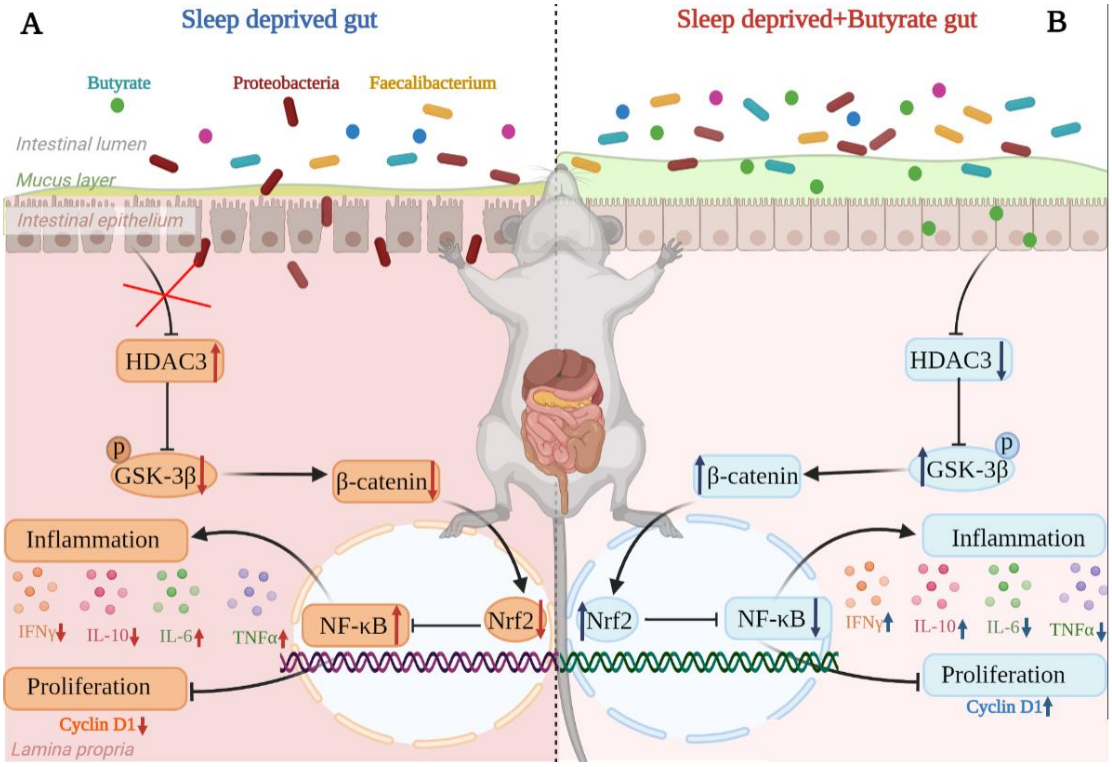

Mechanism of butyrate improving sleep deprivation-induced intestinal mucosal damage in mice. Sleep deficiency caused qualitative and selective change in the gut microbiota composition and decrease the content of *Faecalibacterium* and its metabolite butyrate, ultimately causing small intestinal mucosal injury (A). Further, the pretreatment with butyrate effectively improved the small intestinal mucosal damage through the HDAC3/p-GSK-3β/Nrf2/NF-κB loop in mice (B).

Table S1

Table S1 Investigation of sleep and gastrointestinal condition

1. How many hours do you usually sleep? (   )   [single choice]

Options	Total	Proportion
A. 4-5h	55	10.3%
B. 6-7h	399	74.72%
C. 8-10h	74	13.86%
D. <4h	2	0.37%
E. >10h	4	0.75%
The number of person-times used to fill in the question	534	

2. What do you care most about the sleeping environment? (   )   [multiple choice]

Options	Total	Proportion
A. Light	338	63.3%
B. Temperature	276	51.69%
C. Sound	361	67.6%
D. Comfort of the bed	266	49.81%
The number of person-times used to fill in the question	534	

3. What is your most common posture for sleeping? Which posture is most comfortable for you? (   )   [single choice]

Options	Total	Proportion
A. Lying sideways	331	61.99%
B. Lying down	176	32.96%
C. Lie down	27	5.06%
The number of person-times used to fill in the question	534	

4. When do you feel sleepy or doze off during the day? (   )   [single choice]

Options	Total	Proportion
A. Morning	15	2.81%
B. Noon	404	75.66%
C. Afternoon	94	17.6%
D. Evening	21	3.93%
The number of person-times used to fill in the question	534	

5. What time do you usually go to bed? (Fall asleep time)? (   )   [single choice]

Options	Total	Proportion
A. Before 21 o'clock	6	1.12%
B. 21 to 22 o'clock	30	5.62%
C. 22 to 23 o'clock	120	22.47%
D. After 23:00 o'clock	378	70.79%
The number of person-times used to fill in the question	534	

6. What were you usually doing an hour before going to bed? (   )   [multiple choice]

Options	Total	Proportion
A. Work overtime	77	14.42%
B. Read a book	54	10.11%
C. Watch TV	104	19.48%
D. Play mobile / chat / swipe social software	465	87.08%
E. Watch news / public account	121	22.66%
The number of person-times used to fill in the question	534	

7. How long do you generally need to fall asleep? (   )   [single choice]

Options	Total	Proportion
A. 10 minutes	194	36.33%
B. 10-30 minutes	248	46.44%
C. 30 minutes or more	92	17.23%
The number of person-times used to fill in the question	534	

8. Which of the following questions often plagues you when you fall asleep? (   )   [multiple choice]

Options	Total	Proportion
A. Difficulty falling asleep and not waking up within 30 minutes	169	31.65%
B. Light sleep, easy to wake up	161	30.15%
C. Dreaming so much, I feel dreaming all night	209	39.14%
D. Too much waking at night, feeling insufficient sleep	85	15.92%
E. Wake up prematurely and feel lack of sleep	171	32.02%
The number of person-times used to fill in the question	534	

9. How often do you have insomnia? (   )   [single choice]

Options	Total	Proportion
A. Never	59	11.05%
B. Occasionally	389	72.85%
C. Often	56	10.49%
D. Very often	22	4.12%

E. Always insomnia	8	1.5%
The number of person-times used to fill in the question	534	

10. What causes your insomnia? ( ) [multiple choice]

Options	Total	Proportion
A. Relationship issues (family, friendship, or love)	205	38.39%
B. Heavy workload and overtime	190	35.58%
C. Learning problem	171	32.02%
D. Environmental, medicinal and dietary reasons	95	17.79%
E、Panic, fear, etc.	109	20.41%
F. The sleeping environment is not ideal (such as light, noise, etc.)	226	42.32%
G. Never sleepless	57	10.67%
The number of person-times used to fill in the question	534	

11. How do you wake up in the morning? ( ) [single choice]

Options	Total	Proportion
A. Very good, comfortable and full of energy	66	12.36%
B. Fortunately, it's a relief	206	38.58%
C. No feelings	52	9.74%
D. Still tired and want to sleep	210	39.33%
The number of person-times used to fill in the question	534	

12. How does poor sleep affect you? ( ) [multiple choice]

Options	Total	Proportion

A. No effect	26	4.87%
B. Physical resistance decline	187	35.02%
C. Sleepy during the day	397	74.34%
D. Reduced learning efficiency	285	53.37%
E. Irritability, anxiety, irritability	308	57.68%
F. Memory loss	273	51.12%
G. Acne tease	175	32.77%
The number of person-times used to fill in the question	534	

13. If you have difficulty falling asleep, which of the following measures would you take? ( ) [multiple choice]

Options	Total	Proportion
A. Improve sleep environment	247	46.25%
B. Develop good sleep habits	214	40.07%
C. Relax yourself	332	62.17%
D. Consult a doctor	24	4.49%
E. Strengthen physical exercise	225	42.13%
The number of person-times used to fill in the question	534	

14. Have you felt a heavy stomach after eating in the last week? ( ) [single choice]

Options	Total	Proportion
A. None	287	53.75%
B. Very little	179	33.52%
C. Often feels heavy	63	11.8%
D . Feeling serious and affecting daily life	5	0.94%
The number of person-times used to fill in the question	534	

15. Feeling bloating in the last week? (　　) 　[single choice]

Options	Total	Proportion
A. None	277	51.87%
B. Very little	179	33.52%
C. Often feel bloated	74	13.86%
D . Feeling serious and affecting daily life	4	0.75%
The number of person-times used to fill in the question	534	

16. Have you felt abdominal pain in the last week? (　　) 　[single choice]

Options	Total	Proportion
A. None	348	65.17%
B. Very little	141	26.4%
C. Often feel abdominal pain	41	7.68%
D. Feeling serious and affecting daily life	4	0.75%
The number of person-times used to fill in the question	534	

17. Have you felt abdominal discomfort in the last week? (　　) 　[single choice]

Options	Total	Proportion
A. None	317	59.36%
B. Very little	153	28.65%
C. Often feel abdominal	64	11.99%
D. Feeling serious and affecting daily life	0	0%
The number of person-times used to fill in the question	534	

18. Has there been belching in the past week? (　　) [single choice]

Options	Total	Proportion
A. None	279	52.25%
B. Very little	187	35.02%
C. Often hold your breath after eating	65	12.17%
D. Radon is severe and affects daily life	3	0.56%
The number of person-times used to fill in the question	534	

19. Has there been any venting (fat) in the last week? (　　) [single choice]

Options	Total	Proportion
A. None	71	13.3%
B. Rarely, a little after eating certain foods	252	47.19%
C. Regular exhaust	206	38.58%
D. Feeling serious and affecting daily life	5	0.94%
The number of person-times used to fill in the question	534	

20. Did you feel indigestion in the last week? (　　) [single choice]

Options	Total	Proportion
A. No, it's well digested	259	48.5%
B. Feel a little	223	41.76%
C. Feel more serious	47	8.8%
D. Feeling serious and affecting daily life	5	0.94%
The number of person-times used to fill in the question	534	

21. Total number of bowel movements in the last week: (　　) [single choice]

Options	Total	Proportion
A. 1 times	13	2.43%
B. 2 times	16	3%
C. 3 times	45	8.43%
D. 4 times	38	7.12%
E. 5 times	51	9.55%
F. 6 times	45	8.43%
G. 7 times	157	29.4%
F. 7 or more times	169	31.65%
The number of person-times used to fill in the question	534	

22. Several times of bowel movements in the past week have been laborious: ( ) [single choice]

Options	Total	Proportion
A. 1 times	84	15.73%
B. 2 times	94	17.6%
C. 3 times	36	6.74%
D. 4 or more times	21	3.93%
E. None	299	55.99%
The number of person-times used to fill in the question	534	

23. How many times have I had a bowel movement in the past week? ( ) [single choice]

Options	Total	Proportion
A. 1 times	85	15.92%
B. 2 times	105	19.66%
C. 3 times	62	11.61%

D. 4 or more times	32	5.99%
E. None	250	46.82%
The number of person-times used to fill in the question	534	

24. Have anorectal obstruction a few times every bowel movement in the last week? (   ) [single choice]

Options	Total	Proportion
A. 1 times	83	15.54%
B. 2 times	55	10.3%
C. 3 times	24	4.49%
D. 4 or more times	14	2.62%
E. None	358	67.04%
The number of person-times used to fill in the question	534	

25. How many times do you have a bowel movement in the past week? (   ) [single choice]

Options	Total	Proportion
A. 1 times	7	1.31%
B. 2 times	5	0.94%
C. 3 times	5	0.94%
D. 4 or more times	3	0.56%
E. None	514	96.25%
The number of person-times used to fill in the question	534	

26. Whether you have diarrhea in the last week? (   ) [single choice]

Options	Total	Proportion

A. None	322	60.3%
B. 1-2 times	172	32.21%
C. 3-4 times	29	5.43%
D. 5 or more times	11	2.06%
The number of person-times used to fill in the question	534	

27. Looseness is rare without the use of laxatives (　　) [single choice]

Options	Total	Proportion
A. Yes	234	43.82%
B. No	300	56.18%
The number of person-times used to fill in the question	534	

28. Whether diagnosed with irritable bowel syndrome? (　　) [single choice]

Options	Total	Proportion
A. Yes	31	5.81%
B. No	503	94.19%
The number of person-times used to fill in the question	534	

29. Whether defecation meets one or more of the above-mentioned bowel disorders in the last 3 months? (　　) [single choice]

Options	Total	Proportion
A. Yes	206	38.58%
B. No	328	61.42%
The number of person-times used to fill in the question	534	

30. What are the characteristics of the excreted feces in the last week? Please select your stool

characteristics from the description below? （） [single choice]

Options	Total	Proportion
A. Lumpy, like nuts (difficult to drain)	22	4.12%
B. Sausage-like, but chunky	66	12.36%
C. Sausage-like, but with cracks on the surface	74	13.86%
D. Like snake or sausage, soft and smooth	173	32.4%
E. Soft, sharp-edged blocks	72	13.48%
F. Soft, irregularly edged, mushy stools	124	23.22%
G. Watery, no solid, only liquid	3	0.56%
The number of person-times used to fill in the question	534	

Table S2

Table S2 Quality evaluation of sequencing data

Sample ID	PE Reads	Raw Tags	Clean Tags	Effective Tags	AvgLen(bp)	GC(%)	Q20(%)	Q30(%)	Effective(%)
SD0-1	79822	77697	74480	60283	418	51.62	97.83	95.92	75.52
SD0-2	79963	78106	75451	62754	416	51.53	98.05	96.34	78.48
SD0-3	79714	77842	75104	62979	417	53.64	98.09	96.32	79.01
SD0-4	80222	78433	75817	59262	414	51.95	98.06	96.32	73.87
SD0-5	79700	77800	74872	64609	416	52.21	97.97	96.15	81.07
SD0-6	80148	78355	75408	70249	417	52.46	98.04	96.32	87.65
SD0-7	80047	77696	74250	59549	422	51.17	97.95	96.12	74.39
SD0-8	80010	78396	75868	59568	418	51.46	98.18	96.51	74.45
SD1-1	79992	77269	73539	62230	418	52.46	97.82	95.88	77.8
SD1-2	80056	77452	74150	61040	418	52.43	97.96	96.16	76.25
SD1-3	79889	78051	75660	68924	412	51.57	98.13	96.49	86.27
SD1-4	80055	77577	74620	64699	416	51.85	97.99	96.18	80.82
SD1-5	79909	77324	73935	60026	418	52.01	97.91	96.07	75.12
SD1-6	80438	78291	75511	68435	410	52.26	98.03	96.31	85.08
SD1-7	80086	77109	73353	58699	421	52.23	97.86	95.98	73.29
SD1-8	80087	77881	75072	63731	419	52.72	98.08	96.33	79.58
SD2-1	80075	77221	73676	59050	418	51.2	97.7	95.71	73.74
SD2-2	80091	77379	73953	67759	417	53.23	97.9	96.05	84.6
SD2-3	79993	78013	75205	69405	415	52.68	98.08	96.38	86.76

SD2-4	80262	78304	75411	73443	414	52.91	98.02	96.28	91.5
SD2-5	80487	78071	74922	69144	416	51.27	97.95	96.17	85.91
SD2-6	80117	77571	74467	73995	416	52.06	97.89	96.06	92.36
SD2-7	79731	76949	73354	65279	416	52.33	97.86	96	81.87
SD2-8	79886	77832	75158	59655	417	51.19	98.07	96.35	74.68
SR0-1	79908	76632	72652	65622	423	53.03	97.58	95.47	82.12
SR0-2	79902	77048	73482	67122	420	51.46	97.91	96.08	84.01
SR0-3	68239	66130	63718	55819	416	52.17	97.96	96.14	81.8
SR0-4	79968	77201	73947	69568	424	53.83	97.86	95.9	86.99
SR0-5	79922	77198	73826	59713	419	51.35	97.84	95.95	74.71
SR0-6	79887	77171	74064	61377	415	50.9	97.91	96.08	76.83
SR0-7	80059	76885	72977	61293	421	51.09	97.77	95.84	76.56
SR0-8	80106	78064	75582	62864	414	51.44	98.11	96.41	78.48
SR-1	79913	78142	75457	66680	409	52.74	97.92	96.11	83.44
SR-2	79665	77836	75179	64631	414	51.25	98.08	96.4	81.13
SR-3	79979	77923	74908	63292	421	53.42	98.07	96.27	79.14
SR-4	79869	78325	76022	68107	409	52.76	98.15	96.49	85.27
SR-5	79543	77486	74454	70665	421	54.13	97.92	96.02	88.84
SR-6	80122	77990	74834	59887	420	52.79	97.83	95.92	74.74
SR-7	80047	77719	74568	62313	420	51.47	97.93	96.09	77.85
SR-8	80142	78417	75938	64717	416	52.94	98.2	96.5	80.75
Average	79701.275	77419.65	74371.1	64210.925	417.025	52.18025	97.9615	96.15175	80.56825
Total	3188051	3096786	2974844						
